# Cu‐Pd Dual Single Atoms Promoting Selective CO_2_ Photoreduction to C_2_ Products in Seawater

**DOI:** 10.1002/adma.74420

**Published:** 2026-08-05

**Authors:** Elhussein M. Hashem, Yiran Jiao, Amin Talebian‐Kiakalaieh, Xin Xu, Shiying Ren, Teng Liang, Wenzhong Ji, Teng Lu, Yun Liu, Bingquan Xia, Ashley Slattery, Jingyu Wang, Feiyan Xu, Ping She, Yan Jiao, Jingrun Ran

**Affiliations:** ^1^ School of Chemical Engineering Adelaide University Adelaide Australia; ^2^ State Key Laboratory of Inorganic Synthesis and Preparative Chemistry College of Chemistry International Center of Future Science Jilin University Changchun Jilin China; ^3^ Research School of Chemistry ANU College of Science The Australian National University Canberra Australia; ^4^ Key Laboratory of Green Chemical Engineering Process of Ministry of Education School of Chemistry and Environmental Engineering Wuhan Institute of Technology Wuhan Hubei China; ^5^ Adelaide Microscopy Adelaide University Adelaide Australia; ^6^ Key Laboratory of Material Chemistry for Energy Conversion and Storage (Ministry of Education) School of Chemistry and Chemical Engineering Huazhong University of Science and Technology Wuhan China; ^7^ Laboratory of Solar Fuel Faculty of Materials Science and Chemistry China University of Geosciences Wuhan China

**Keywords:** C_2_ fuels, CO_2_ photoreduction, dual single atom catalyst, photocatalysis, seawater

## Abstract

The solar‐powered CO_2_ conversion via the photocatalysis route offers a sustainable pathway toward carbon neutrality while mitigating energy/environmental pressure. Nevertheless, the selective and efficient conversion of CO_2_ via photoreduction to C_2_ products remains a formidable challenge. Here, we engineered a dual‐single‐atom photocatalyst by controllably embedding Pd and Cu single atoms into a TiO_2_ matrix. The optimized catalyst (Cu_0.5_Pd_0.5_/TiO_2_) exhibits the outstanding yield (119.2 µmol/g_cat_) and selectivity (84.8%) for acetic acid production from CO_2_ photoreduction, performed in seawater and in a photothermal‐aided reactor. Various in situ/ex situ characterizations were employed to investigate atomic‐level structure‐performance correlation and reaction mechanism in practical condition. In situ x‐ray photoelectron spectroscopy, in situ atomic force microscopy‐Kelvin probe force microscopy, transient‐state surface photovoltage, and in situ electron paramagnetic resonance (EPR) collectively indicate that loading Pd and Cu single atoms onto TiO_2_ apparently accelerates charge kinetics. This modification results in increased photogenerated electrons for CO_2_ reduction, facilitating C─C coupling and hydrogenation reactions. Additionally, in situ infrared (IR) spectroscopy and theoretical computations affirm the pivotal function of Pd single atoms for lowering the energy barrier to form the ^*^OCCO intermediate, apparently improving selectivity for acetic acid production. Overall, our work presents an innovative approach to tackle kinetic and thermodynamic challenges for light‐induced CO_2_‐to‐C_2_ conversion.

## Graphical abstract

Cu and Pd dual single atoms anchored on TiO_2_ cooperatively enhance C─C coupling during CO_2_ photoreduction in seawater. Under xenon‐light irradiation and without a sacrificial reagent/alkaline trapping agent, the catalyst achieves highly selective acetic acid formation. The cooperative dual‐single‐atom configuration provides an efficient pathway for sustainable solar‐to‐chemical conversion of CO_2_ into C_2_ products.

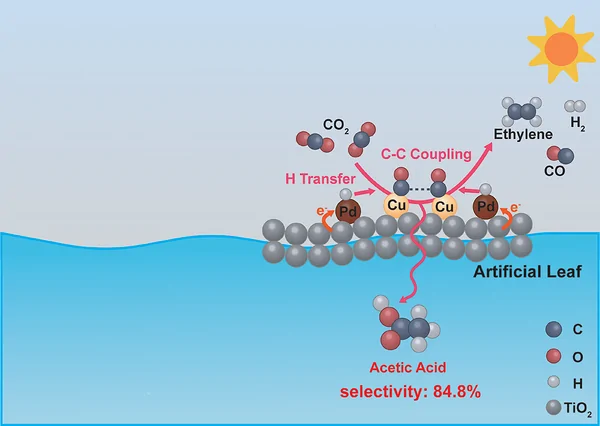

## 1 Introduction

Photocatalytic reduction of carbon dioxide (CO_2_) into solar fuels has gained tremendous attention over the past decade, both in academic research and industrial applications. This approach represents a cutting‐edge strategy for advancing carbon neutrality and exploiting sunlight to facilitate CO_2_ conversion into valuable hydrocarbons [1, 2, 3, 4, 5, 6]. Despite notable advances in CO_2_ photoreduction (CO_2_ PR) technique, most current systems predominantly yield C_1_ products, including carbon monoxide (CO) [7, 8, 9], methane (CH_4_) [9, 10, 11], formic acid (HCOOH) [12, 13, 14], and methanol (CH_3_OH) [15, 16, 17]. This limitation tremendously hampers their potential for practical applications [18, 19]. The production of higher‐value C_2_ products, for example, ethanol, acetic acid (CH_3_COOH), and ethylene (C_2_H_4_), remains elusive, primarily owing to challenges related with sluggish reaction kinetics, complex multi‐electron/proton transfers, and thermodynamic energy barriers in C─C coupling mechanism. Hence, this results in lower yields and selectivity for the sought‐after C_2_ products [20, 21, 22, 23]. Many current works focusing on C_2_ product preparation utilize toxic/costly reagents, for example, triethanolamine as a sacrificial agent, introducing tremendous environmental concerns related to waste management [24, 25, 26]. Thus, there is an urgent need to develop efficient photocatalysts that can effectively facilitate multi‐electron transfer for CO_2_ PR to generate C_2_ products while achieving high selectivity [27, 28]. This advancement is crucial to meet the growing global demand for sustainable generation of C_2_ products [29, 30].

The main challenges in CO_2_‐to‐C_2_ conversion originate from the inherent inertness of CO_2_ molecules, resulting in high energy barriers for effective adsorption/activation of CO_2_. Catalysts are required to realize the following functions consecutively: (i) facilitating the formation of C_1_ intermediates; (ii) alleviating the electrostatic repulsion between these intermediates to enhance their collision probability; (iii) combining these intermediates to ultimately yield the desired C_2_ products upon desorption [31, 32, 33]. Furthermore, the complex multi‐electron/‐proton transfer mechanism in the reduction reactions to produce C_2_ products, the ability of generating specific oxygenated radicals (e.g., hydroxyl radicals) and the thermodynamics barriers for the coupling of produced C_1_ intermediates [34, 35, 36] contribute to reduced product yields and selectivity [37, 38, 39]. Thus, the single‐atom photocatalysts, particularly those based on copper, have been introduced to address these challenges, particularly in achieving the right balance between multi‐electron and proton transfers, improving charge separation, and overcoming thermodynamic barriers associated with C─C coupling [8, 40, 41]. This balance is critical for enhancing the activity and selectivity for C_2_ products [34, 42, 43]. Copper (Cu)‐based dual‐single‐atom photocatalysts have been developed to enhance the efficiency of CO_2_ PR to C_2_ products further. In this approach, Cu sites act as reaction coupling points, while adjacent sites promote electron transfer, leading to significant improvements in the activity and selectivity of C_2_ hydrocarbons [44, 45, 46, 47]. Additionally, utilizing seawater as a medium improves photocatalytic performance due to its electrolytic content, which increases ionic strength and conductivity for efficient carrier separation and redox reactions [48, 49, 50, 51]. The design of artificial leaves, featuring a triple‐phase junction (gas‐liquid‐solid), facilitates CO_2_ diffusion, proton transfer, and charge separation, optimizing light harvesting and product selectivity [52, 53]. These innovations are promising for scaling up CO_2_ reduction for real‐world applications, although reports on their integration are limited.

Furthermore, despite the tremendous advances of CO_2_‐to‐C_2_ conversion via photocatalysis route in recent years [54, 55, 56, 57, 58], the charge carrier separation/transport/trapping/releasing kinetics within photocatalyst and the reaction pathways on the surface of photocatalyst, particularly in the realistic reaction condition (e.g., light irradiation, CO_2_ atmosphere, in certain solvent and at specific temperature), remain elusive and unclear [59]. To explicitly unravel the above‐mentioned key information in the complex photocatalytic process, state‐of‐art in situ characterization techniques are urgently required to be employed. For example, in situ x‐ray photoelectron spectroscopy (XPS), in situ electron paramagnetic resonance (EPR) spectroscopy and in situ atomic force microscopy coupled with Kelvin probe force microscopy (AFM‐KPFM) can provide the important information of element chemical status on/near the surface of catalyst in the depth of several nanometers, the (quasi‐)quantitative concentration of defects/certain metal ions within the catalyst in the depth of tens to hundreds of micrometers, and space‐resolved surface potential on/near the surface of catalyst in the depth of several nanometers, respectively, in certain conditions (e.g., under light irradiation, in CO_2_/O_2_/air/argon atmosphere, at elevated temperature and in the presence of specified chemical reactant) [60, 61]. The above uncovered information can further reveal the element‐resolved/species‐resolved/space‐resolved electron or hole separation/migration/trapping/releasing on the very surface of the photocatalyst or within the bulk of photocatalyst [62]. Additionally, in situ EPR spectroscopy and in situ infrared (IR) spectroscopy can unravel the time‐resolved information of radicals in the solution and adsorbed intermediates on the catalyst surface to clarify the precise reaction pathway at the molecular level [63]. Most interestingly, the comprehensive analyses and understanding of all the above in situ characterization results can present a complete or much clearer picture regarding the three consecutive processes (i.e., charge generation, charge separation/transfer, charge‐involved redox reactions) within the bulk and on the surface of photocatalyst in desired conditions, which are considered as “black box” information in the past. Unfortunately, the comprehensive application of all the above in situ characterization tools and their combination with theoretical computations, are not often reported, for the photocatalytic CO_2_‐to‐C_2_ conversion.

**FIGURE 1:**
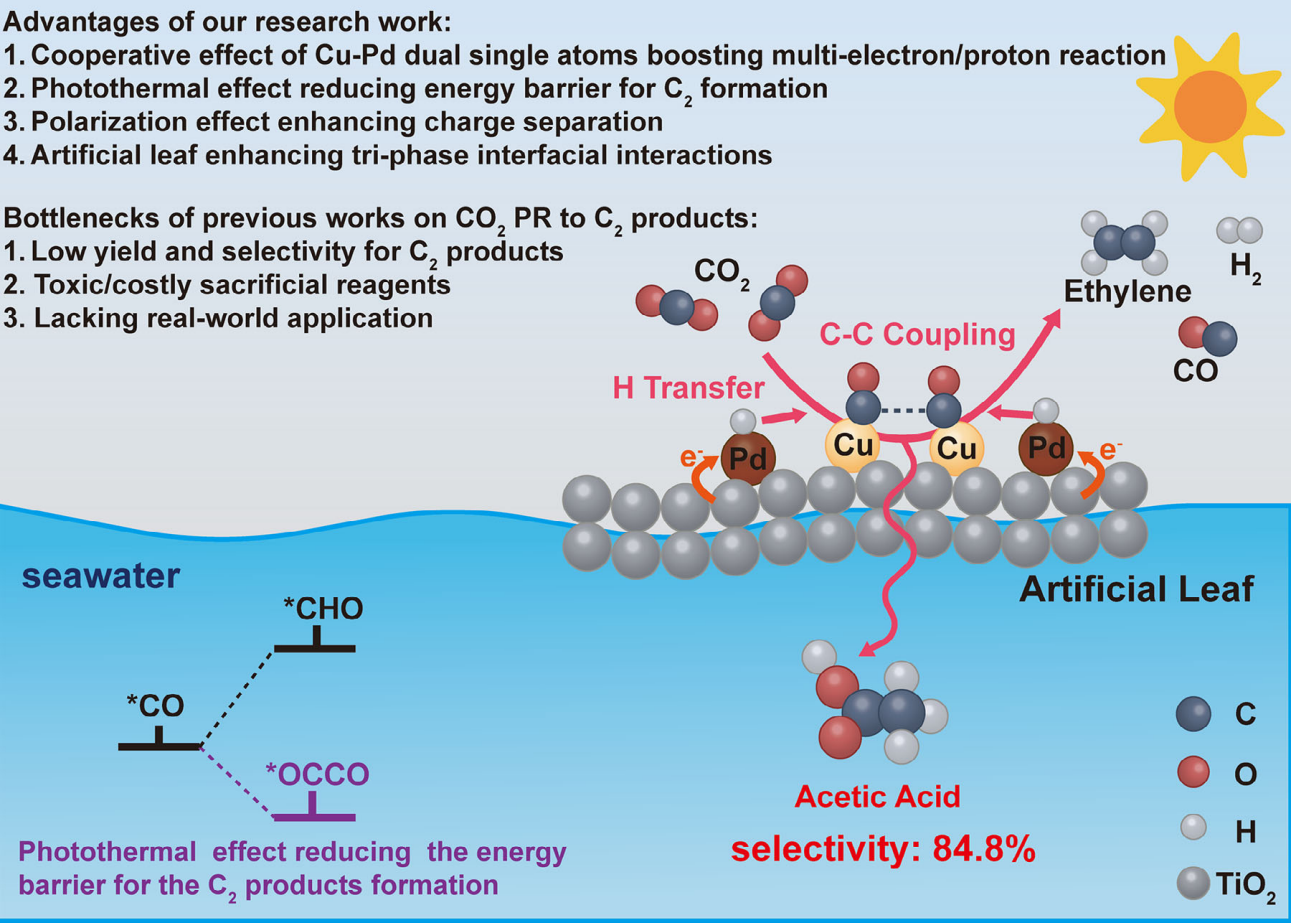
Overview of the floatable artificial leaf setup for the CO_2_ PR into valuable C_2_ products by utilizing the optimized Cu_0.5_Pd_0.5_/TiO_2_ photocatalyst.

In this study, we report the preparation of a novel dual‐single‐atom Cu‐Pd/TiO_2_ catalyst for enhanced CO_2_ PR utilizing seawater. The optimized catalyst (Cu_0.5_Pd_0.5_/TiO_2_) exhibits an impressive production of CH_3_COOH (119.2 µmol/g), without any sacrificial agent, after 6‐hour full‐spectrum light irradiation and at an elevated reaction temperature (65°C) induced by photothermal effect. Remarkably, this catalyst exhibits a 100% CH_3_COOH selectivity as the only liquid product, resulting in an overall selectivity of 84.8% for CH_3_COOH relative to the total solar fuels produced. Additionally, after extending reaction time to 12 h, a total generation of 2.6 µmol/g C_2_H_4_ is achieved. The control experiments results indicate that the observed products originated from CO_2_ photoreduction. Furthermore, a floatable artificial leaf was engineered to replicate realistic reaction conditions, resulting in a production of C_2_H_4_ (3.1 µmol/g) and CH_3_COOH (66.4 µmol/g) after 24‐hour light irradiation. The incorporated Pd SAs facilitates the electron/proton transfer to the Cu sites, where the C─C coupling occurs, resulting in a synergistic effect lowering the thermodynamic energy barriers associated with the C_2_ products generation. Theoretical calculations indicate that adding Pd SAs to the Cu SAs matrix reduces the energy barrier for the formation of key intermediate ^*^OCCO in C─C coupling process. A range of in situ/ex situ advanced characterizations and theoretical calculations were employed to elucidate atomic‐level structure‐performance relationship and provide valuable insights into reaction mechanisms. In situ spectroscopic techniques, including XPS, AFM‐KPFM, transient‐state surface photovoltage (TPV), and EPR, revealed that coupling of Pd SAs and Cu SAs enhances charge separation. This modification reduces overall recombination rate and facilitates multi‐electron transfer, tremendously promoting C─C coupling processes. Additionally, in situ transient‐state photoluminescence (TP) spectroscopy reveals that seawater utilized as a reaction medium boosts charge separation/transfer, effectively extending carrier lifetimes. Furthermore, in situ IR spectroscopy provides insights into the reaction mechanism and identifies the key intermediates (e.g., ^*^OCCO and ^*^CH_3_COOH) responsible for generating the targeted C_2_ products. These results highlight the potential of a dual‐single‐atom catalyst for scaling up CO_2_ photoreduction to generate C_2_ products in realistic application. The schematic image is exhibited in Figure 1.

## 2 Results and Discussion

### 2.1 Catalyst Composition and Microstructure

A range of modified Cu (Cu SAs), palladium (Pd SAs), and copper/palladium (Cu‐Pd dual single atoms) supported on TiO_2_ photocatalysts were respectively prepared utilizing the wet impregnation technique, with complete experimental procedures recorded in the Supporting Information. Initially, the loading amount of Cu SAs on TiO_2_ was optimized. Then, various loadings of Pd SAs were incorporated to generate the Cu‐Pd dual‐single‐atom loaded TiO_2_ catalyst (Cu‐Pd/TiO_2_). For comparative analysis, a catalyst with identical Pd loading was also prepared. According to the weight ratios for Cu and Pd elements, the prepared catalysts were labelled Cu_0.25_/TiO_2_, Cu_0.5_/TiO_2_, Cu_0.75_/TiO_2_, Cu_1_/TiO_2_, Cu_0.5_Pd_0.2_/TiO_2_, Cu_0.5_Pd_0.4_/TiO_2_, Cu_0.5_Pd_0.5_/TiO_2_, and Pd_0.5_/TiO_2_, respectively. Figure S1 exhibits the x‐ray diffraction (XRD) patterns of pure TiO_2_, as‐prepared photocatalysts, along with the JCPDS cards of anatase (#21‐1272) and rutile (#21‐1276), respectively. The XRD analysis demonstrates that all prepared photocatalysts exhibit distinct diffraction peaks for rutile and anatase phases, with no detectable signatures for Cu or Pd species. These results indicate that both Cu and Pd species are well‐dispersed on the TiO_2_ surface at low concentrations. The microstructure and morphological of the optimized photocatalysts (Cu_0.5_Pd_0.5_/TiO_2_) were examined using transmission electron microscopy (TEM) and high‐angle annular dark field‐scanning transmission electron microscopy (HAADF‐STEM). Figure S2a presents the TEM image of the optimized Cu SAs/TiO_2_ catalyst (Cu_0.5_/TiO_2_), which reveals the absence of Cu nanoparticles/clusters/ agglomerates. Figure S2b,c display the HAADF‐STEM and atomic‐resolution HAADF‐STEM images of Cu_0.5_/TiO_2_ catalyst, respectively, confirming the presence of isolated Cu single atoms (SAs) as bright dots on the surface of TiO_2_. Additionally, the HAADF‐STEM image (Figure S2d) and the corresponding elemental mapping images (Figure S2e–g) of Cu_0.5_/TiO_2_ catalyst further corroborate the uniform distribution of Cu atoms throughout the TiO_2_ support. Furthermore, the TEM images (Figure S3a–d) of the optimized catalyst (Cu_0.5_Pd_0.5_/TiO_2_) exhibit no visible clustering of Cu/Pd elements, also implying the successful loading of Cu/Pd dual single atoms on TiO_2_ catalyst.

**FIGURE 2:**
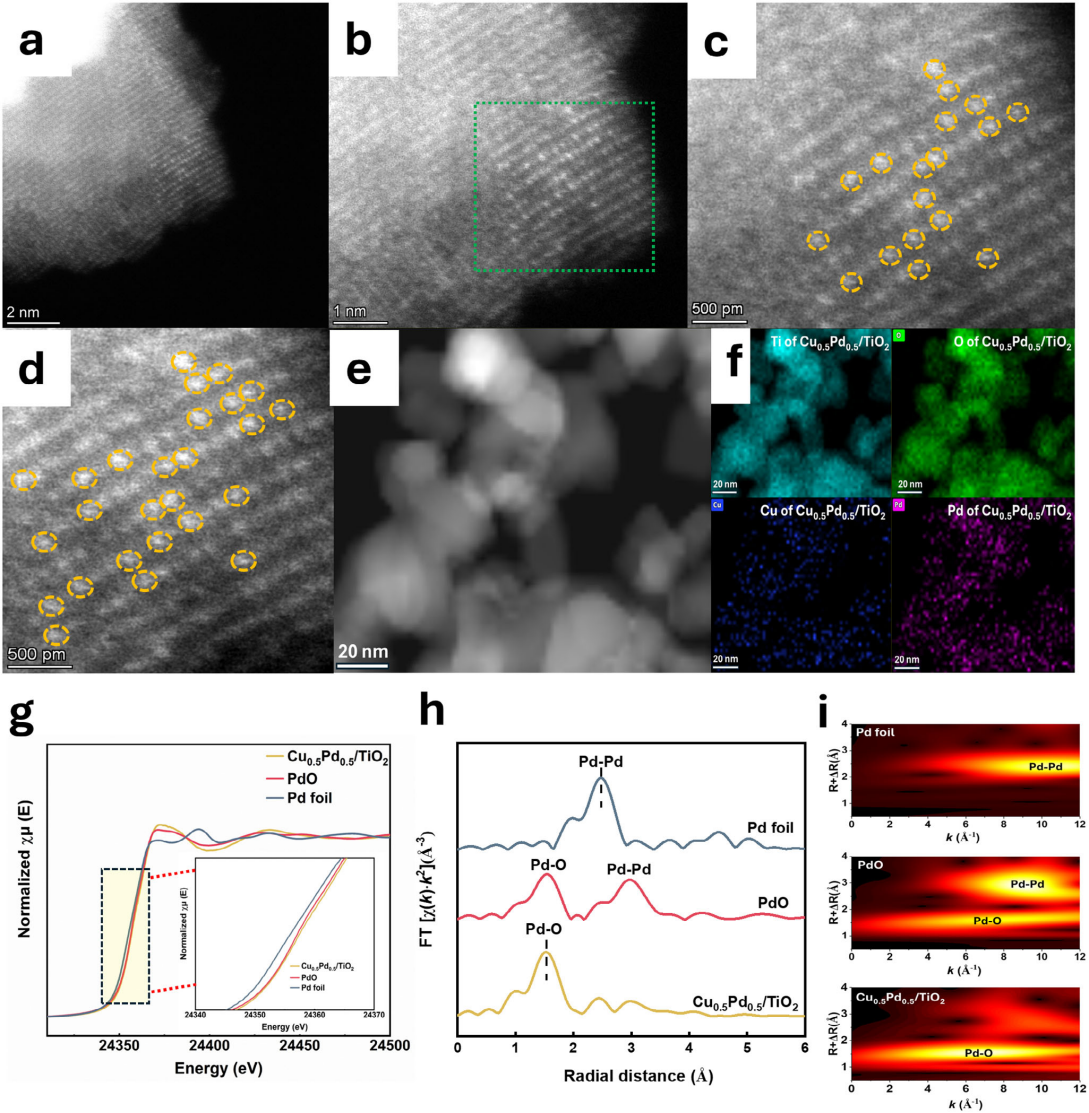
(a,b) HAADF‐STEM images of the optimized Cu_0.5_Pd_0.5_/TiO_2_. (c,d) Atomic‐Resolution HAADF‐STEM images of Cu_0.5_Pd_0.5_/TiO_2_ from the green rectangle region in Figure 2b. (e) HAADF‐STEM image of Cu_0.5_Pd_0.5_/TiO_2_ and (f) the corresponding elemental mapping images of Ti, O, Cu, and Pd elements for Cu_0.5_Pd_0.5_/TiO_2_. (g) The synchrotron‐based XANES spectra for Pd K edge of Cu_0.5_Pd_0.5_/TiO_2_, Pd foil, and PdO, respectively. Figure 2g inset exhibits the near‐edge features for XANES spectra of Cu_0.5_Pd_0.5_/TiO_2_, Pd foil, and PdO, respectively. (h) Fourier‐transformed extended x‐ray absorption fine structure (EXAFS) spectra for Pd foil, PdO, and Cu_0.5_Pd_0.5_/TiO_2_, respectively. (i) Pd K‐edge WT‐EXAFS spectra for Cu_0.5_Pd_0.5_/TiO_2_, PdO, and Pd foil, respectively.

The HAADF‐STEM images of Cu_0.5_Pd_0.5_/TiO_2_ (Figure 2a,b) imply a uniform distribution of Pd/Cu atomic sites, observed as scattered bright spots across the TiO_2_ surface. The green rectangle region highlighted in Figure 2b was further analyzed with atomic‐resolution HAADF‐STEM and the results in Figure 2c,d reveal the excellent dispersion of Pd/Cu atoms, with the absence of any large particle aggregation on the surface of Cu_0_._5_Pd_0_._5_/TiO_2_ catalyst.

The intensity profiles of HAADF‐STEM image are crucial for differentiating isolated Cu and Pd SAs on a TiO_2_ support. This is because that HAADF‐STEM image intensity is intimately related to element atomic number, with apparent z‐contrast. The intensity of HAADF‐STEM image adheres to the relationship: (I_HAADF_ ∝ Z^1.5−1.8^) [64]. Therefore, Pd atoms, scattering electrons more effectively than Cu atoms, exhibit an apparently brighter appearance on a TiO_2_ background. This relationship can be quantitatively expressed as:

$$\frac{I_{\mathrm{Pd}}}{I_{\mathrm{Cu}}}\approx {\left(\frac{46}{29}\right)}^{1.7}\approx 2.2$$

As a result, isolated Pd atoms can appear approximately twice as bright as their Cu counterparts under similar imaging conditions. As exhibited in Figure S4a,b, we can find the existence of one Pd single atom along with three Cu single atoms. Additionally, the HAADF‐STEM image (Figure 2e), along with the elemental mapping results (Figure 2f), corroborate the homogeneous distribution of Pd and Cu atomic sites. All the above results imply the highly dispersion of Pd‐Cu dual single atoms on the surface of TiO_2_ nanoparticles in Cu_0.5_Pd_0.5_/TiO_2_ catalyst.

Furthermore, inductively coupled plasma‐mass spectrometry (ICP‐MS) results reveal the presence of 0.495 wt% Cu element and 0.471 wt% Pd element in Cu_0.5_Pd_0.5_/TiO_2_ catalyst. The low loading amounts of both Cu and Pd elements in Cu_0.5_Pd_0.5_/TiO_2_ catalyst also imply the uniform distribution of Cu and Pd atoms on the surface of catalyst. To gain deeper insight into the electronic environment and local coordination of both metals, synchrotron‐based x‐ray absorption spectroscopy (XAS) was performed. As exhibited in Figure S5a, the synchrotron‐based x‐ray absorption near‐edge structure (XANES) spectra for the Cu K‐edge of Cu_0.5_/TiO_2_ (before incorporating Pd single atoms) was examined and compared with various reference standards, including Cu foil, Cu_2_O, and CuO. The positioning of the main absorption edge and peaks for Cu_0.5_/TiO_2_ closely aligns with those of CuO, in apparent contrast to the edge and peaks of Cu foil and Cu_2_O. These results indicate that the valence state of Cu SAs in this matrix is ∼+2. As exhibited in Figure S5b, the Fourier transform k^2^‐weighted extended x‐ray absorption fine structure (FT‐EXAFS) spectra in R space for Cu_2_O, CuO, and Cu_0.5_/TiO_2_ all exhibit a prominent peak at ∼1.5 Å, attributed to Cu‐O coordination [65]. Notably, the absence of a Cu‐Cu peak at ∼2.2 Å for Cu_0.5_/TiO_2_ (Figure S5b) confirms the atomic dispersion of Cu species. The wavelet‐transform (WT) analysis of EXAFS spectra for Cu_0.5_/TiO_2_ indicates a damped intensity maximum at ∼5.8 Å^−^^1^ (Figure S5c), reflecting the contribution of Cu─O bond [66]. As exhibited in Figure S5c, the lack of Cu─Cu coordination for Cu_0.5_/TiO_2_, as evidenced by comparison to Cu foil, Cu_2_O, and CuO references, clearly confirms the atomically dispersed Cu species in Cu_0.5_/TiO_2_ [67]. Additionally, Figure S6a exhibits the XANES spectra for the Cu K‐edge of Cu_0.5_Pd_0.5_/TiO_2_ after loading the Pd SAs onto the previously prepared Cu_0.5_/TiO_2_ catalyst, along with Cu Foil, Cu_2_O, and CuO as references. The comparison between the Cu‐K edge XANES spectra results for Cu_0.5_/TiO_2_ (Figure S5a) and Cu_0.5_Pd_0.5_/TiO_2_ (Figure S6a) catalysts indicate their similar absorption edge and main peak positions, which are also close to those of the references CuO in Figures S5a and S6a, respectively. These results clearly confirm that the valence state of Cu SAs (+2) in Cu_0.5_Pd_0.5_/TiO_2_ remains unchanged after loading the Pd SAs onto Cu_0.5_/TiO_2_. Further analysis of the FT‐EXAFS spectra in R space for Cu_2_O, CuO, and Cu_0.5_Pd_0.5_/TiO_2_ (Figure S6b) also reveal an apparent peak at ∼1.5 Å, attributed to Cu‐O coordination. Furthermore, the absence of a Cu‐Cu peak at ∼2.2 Å in Cu_0.5_Pd_0.5_/TiO_2_ confirms the atomic dispersion of Cu species following the addition of Pd SAs. Finally, the WT‐EXAFS results of Cu_0.5_Pd_0.5_/TiO_2_ (Figure S6c) further corroborate the existence of Cu‐O interactions while confirming the absence of Cu‐Cu coordination. These findings collectively affirm the isolated nature of the incorporated Cu single atoms in the optimized Cu_0.5_Pd_0.5_/TiO_2_ matrix.

Furthermore, Figure 2g exhibits the synchrotron‐based XANES Pd K‐edge spectra of Cu_0.5_Pd_0.5_/TiO_2_, compared with those of PdO and Pd foil references. The absorption edge in the XANES Pd K‐edge spectra of Cu_0.5_Pd_0.5_/TiO_2_ (Figure 2g) indicates the position close to that of standard PdO, confirming that the Pd element of Cu_0.5_Pd_0.5_/TiO_2_ is in the valence state of ∼+2 [68]. Moreover, the FT‐EXAFS spectra in R space for Cu_0.5_Pd_0.5_/TiO_2_ (Figure 2h) exhibits a singular peak at 1.53 Å, corresponding to the Pd‐O coordination shell of PdO. There is weak scattering observed around 2.5 Å, probably arising from second‐shell coordination, support‐mediated backscattering pathways, and/or multiple‐scattering contributions [69, 70]. Additionally, the FT‐EXAFS spectra in R space for Cu_0.5_Pd_0.5_/TiO_2_ (Figure 2h) reveal no evidence of Pd‐Pd interactions, clearly revealing the formation of isolated Pd SAs coordinated via Pd─O bonding within the matrix [71]. The corresponding WT‐EXAFS analysis (Figure 2i) for Cu_0.5_Pd_0.5_/TiO_2_ exhibits a distinct intensity maximum at ∼6.9 Å^−^^1^, attributed to the Pd─O bonding in the first nearest‐neighbor coordination shell. Meanwhile, no maxima at 9.5 Å^−^^1^ relevant to Pd‐Pd interactions are detected for Cu_0.5_Pd_0.5_/TiO_2_, as revealed in Figure 2i. The above results indicate that Pd SAs are effectively anchored onto TiO_2_ through Pd─O bonding [72]. Collectively, these results indicate that the Cu and Pd species within the Cu_0.5_Pd_0.5_/TiO_2_ system predominantly exist as isolated O‐coordinated atomic sites or Pd‐Cu dual single atoms.

Furthermore, x‐ray photoelectron spectroscopy (XPS) technique was utilized to provide insights into the chemical states of Cu and Pd SAs and their interactions with TiO_2_. As exhibited in Figure S7a,b, the Ti 2p and O 1s peaks for Cu_0.5_Pd_0.5_/TiO_2_ are moved to the higher binding energy direction, compared to those for TiO_2_. These peak movements reveal the electron migration from TiO_2_ to loaded Cu/Pd SAs [73]. Figure S7c exhibits the Cu 2p spectra for Cu_0.5_Pd_0.5_/TiO_2_, revealing two distinct peaks at 932.3 and 952.4 eV, corresponding to the Cu^+^ state. It is essential to note that Cu^+2^ species can be readily reduced to Cu^+^ under high‐vacuum conditions owing to the x‐ray irradiation bombardment effect, particularly when the content of highly dispersed Cu is minimal [65]. Moreover, the Pd 3d spectrum for Cu_0.5_Pd_0.5_/TiO_2_ (Figure S7d) exhibits two peaks at 336.3 and 341.7 eV, attributed to the Pd^+2^ oxidation state [74]. To clearly confirm the oxidation state of Cu element in the Cu_0.5_Pd_0.5_/TiO_2_ composite, the EPR technique was applied. As exhibited in Figure S8, the spectrum of Cu_0.5_Pd_0.5_/TiO_2_ presents a prominent peak at g = 2.05, indicative of paramagnetic Cu^2^^+^ species, well matched with the XANES spectra results (Figure S6a) [75].

Although the ICP‐MS measurements indicate similar bulk loadings of Cu and Pd, the quality of XPS spectra is not determined only by elemental concentration. For atomically dispersed metal species, the detected XPS intensity is intimately affected by surface distribution, local coordination environment, photoionization cross‐sections, intrinsic sensitivity factors, and background characteristics of the corresponding spectral region. In particular, the Cu 2p region is often complicated by broad line shapes, satellite features, and a relatively high background, which can substantially reduce the signal‐to‐noise ratio for highly dispersed Cu species. By comparison, the Pd 3d region generally exhibits a cleaner background and narrower spectral features, resulting in a more readily interpretable spectrum under identical acquisition conditions. Therefore, we consider the Cu 2p spectrum as supplementary evidence rather than the primary basis for Cu speciation. Theoretically, Cu LMM Auger spectroscopy can provide complementary information for Cu oxidation‐state determination. Nevertheless, owing to the extremely low loading and atomically dispersed nature of Cu in Cu_0.5_Pd_0.5_/TiO_2_, reliable Cu LMM Auger spectra with sufficient signal‐to‐noise ratio cannot be acquired. As a result, the chemical state and local coordination environment of Cu were primarily determined utilizing Cu K‐edge XANES, EXAFS, WT‐EXAFS, and EPR spectroscopy, which are extensively recognized as more appropriate techniques for characterizing highly dispersed Cu single‐atom species. To further improve the structural assignment, we have provided quantitative EXAFS fitting results for Cu_0.5_Pd_0.5_/TiO_2_ (Table S1). The fitting reveals a Cu─O coordination number (CN) of ∼3 and a Cu─O bond distance of 1.97 ± 0.01 Å, while no detectable Cu─Cu scattering contribution is observed. These results confirm that Cu species are atomically dispersed and predominantly coordinated with oxygen atoms on the TiO_2_ surface. The afore‐mentioned results strongly corroborate the successful preparation of Pd‐Cu dual‐single‐atom loaded TiO_2_ nanoparticles (Cu_0.5_Pd_0.5_/TiO_2_).

### 2.2 Photocatalytic Performance for CO_2_ Photoreduction (PR) in Seawater

**FIGURE 3:**
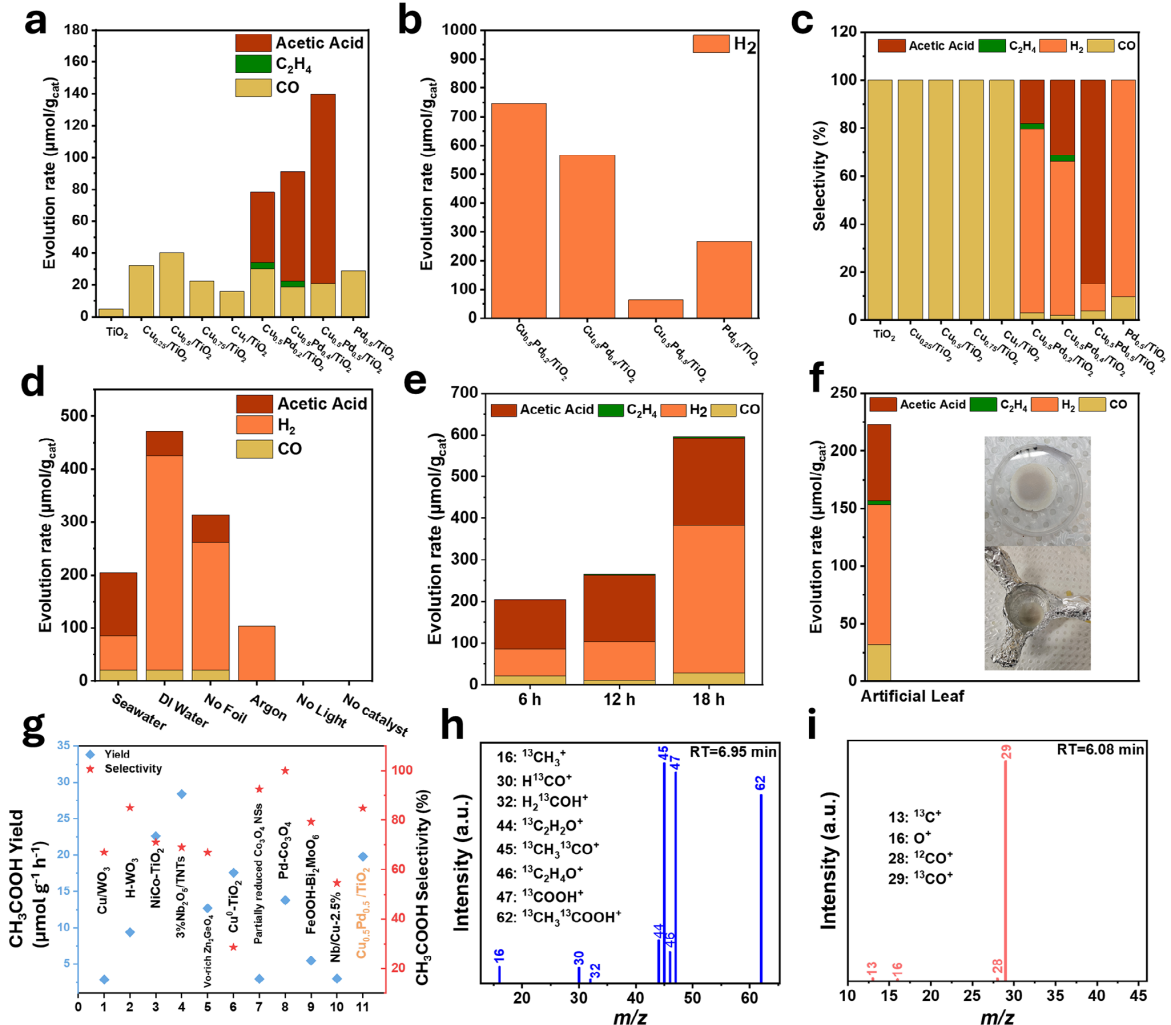
(a) Carbon‐based products evolution rates for the as‐prepared photocatalysts after 6‐h reaction under light irradiation in seawater. (b) Hydrogen (H_2_) evolution rates for Cu_0.5_Pd_0.2_/TiO_2_, Cu_0.5_Pd_0.4_/TiO_2_, Cu_0.5_Pd_0.5_/TiO_2_ and Pd_0.5_/TiO_2_ catalysts after 6‐h reaction under light irradiation in seawater, respectively. (c) The selectivity of each product for all the catalysts after 6‐h reaction in seawater. (d) Control experiments for Cu_0.5_Pd_0.5_/TiO_2_. (e) The robustness test results of Cu_0.5_Pd_0.5_/TiO_2_ for three consecutive cycles in seawater. (f) Artificial leaf products yield after 24‐h xenon light irradiation in seawater. (g) A comparison of photocatalytic CO_2_ reduction performances for acetic acid yield and selectivity in the reported references and by the optimized Cu_0.5_Pd_0.5_/TiO_2_ [54, 76, 77, 78, 79, 80, 81, 82, 83, 84]. (h) Mass spectrum of the produced (^13^CH_3_^13^COOH) after ^13^CO_2_ photoreduction over Cu_0.5_Pd_0.5_/TiO_2_. (i) Mass spectrum of produced (^13^CO_2_) after ^13^CO_2_ photoreduction over Cu_0.5_Pd_0.5_/TiO_2_.

Then, we have systematically tested the catalytic performances of all the as‐prepared catalysts for light‐driven CO_2_ PR in seawater, operating without any sacrificial agent. A layer of aluminium foil was applied to cover the outside surfaces of the reactor, exposing only the top surface for light irradiation, which can optimize light absorption/harvesting. This setup harnesses light as the only energy resource, with efficient IR light utilization, which can raise and retain the temperature inside the reactor at ∼65°C to greatly boost the catalytic reaction kinetics and improve the overall photocatalytic performances. This increased temperature was found to obviously promote the C─C coupling mechanism, which was confirmed by experimental results in the later discussion. Before the reaction, we filtered the collected seawater to remove sand, microorganisms, and other impurities, then analyzed it using ^1^H NMR technique to ensure no compounds interfered with the catalytic process (Figure S9). Furthermore, we have conducted an ion analysis of the collected seawater to analyze its ion contents and concentrations, as exhibited in Table S2. Figure 3a exhibits the yields of carbon‐based products from CO_2_ photoreduction (PR) across various photocatalysts after a 6‐hour reaction period. The results (Figure 3a) reveal that loading Cu SAs onto TiO_2_ boosts CO production, increasing the evolved amount from 5.1 µmol/g_cat_ for bare TiO_2_ to 32.5 µmol/g_cat_ for Cu_0.25_/TiO_2_. The highest yield is observed over Cu_0.5_/TiO_2_, producing 40.3 µmol/g_cat_ of CO. However, increasing Cu concentration further lowered photocatalytic efficiency, with Cu_0.75_/TiO_2_ and Cu_1_/TiO_2_ yielding only 22.5 and 16.1 µmol/g_cat_ of CO, respectively. Interestingly, further loading Pd SAs onto the optimized Cu_0.5_/TiO_2_ catalyst results in the formation of C_2_ products together with CO. The Cu_0.5_Pd_0.2_/TiO_2_ catalyst exhibits a yield of 4.03 µmol/g_cat_ for C_2_H_4_ and 44.1 µmol/g_cat_ for CH_3_COOH, in addition to 30.04 µmol/g_cat_ of CO. As the loading amount of Pd SAs is increased from 0.2% to 0.5 wt%, a significant rise in acetic acid (CH_3_COOH) production is perceived. The Cu_0.5_Pd_0.4_/TiO_2_ catalyst yields 3.8 µmol/gcat of C_2_H_4_, 69.05 µmol/g_cat_ of CH_3_COOH, and 18.6 µmol/g_cat_ of CO. Particularly, Cu_0.5_Pd_0.5_/TiO_2_ reaches the maximum production with 119.2 µmol/g_cat_ of CH_3_COOH and 20.85 µmol/g_cat_ of CO. In contrast, the Pd_0.5_/TiO_2_ catalyst produced only CO, yielding 28.88 µmol/g_cat_ of CO gas. These findings highlight the important role of Pd SAs in facilitating C─C coupling processes and producing C_2_ products.

To precisely test the components of all the produced liquid products, a range of techniques, including proton nuclear magnetic resonance (^1^H NMR), gas chromatography‐mass spectrometry (GC‐MS), and high‐performance liquid chromatography (HPLC), were employed.

The corresponding results are exhibited in Figures S10a–c, S11a–d, and S12a–c, respectively. All the above results confirm the presence of acetic acid as the only liquid product.

Notably, the anchoring of Pd SAs onto the Cu_0.5_/TiO_2_ catalyst also results in H_2_ evolution, as exhibited in Figure 3b. Compared to Pd_0.5_/TiO_2_ (266.64 µmol/g_cat_), Cu_0.5_Pd_0.2_/TiO_2_, Cu_0.5_Pd_0.4_/TiO_2_ and Cu_0.5_Pd_0.5_/TiO_2_ present the H_2_ production of 746 µmol/g_cat_, 566.5 and 64.7 µmol/g_cat_, respectively. These results (Figure 3b) clearly reveal that loading Pd SAs enhances hydrogenation and shifts yields toward CH_3_COOH. Figure 3c exhibits the selectivity for each product generated by the as‐prepared photocatalysts. For various Cu loaded TiO_2_ (Cu_0.25_/TiO_2_, Cu_0.5_/TiO_2_, Cu_0.75_/TiO_2_ and Cu_1_/TiO_2_), the selectivity of CO reaches 100%. These results indicate that the loaded Cu species alone in this system can only promote CO_2_‐to‐CO conversion. In contrast, the loading of Pd imposes the significant impact on the product selectivity: (i) Cu_0.5_Pd_0.2_/TiO_2_ exhibits the selectivity of 3.1% for CO, 2.4% for C_2_H_4_, 18.1% for CH_3_COOH, and 76.4% for H_2_, respectively. (ii) Cu_0.5_Pd_0.4_/TiO_2_ presents the selectivity of 2.1% for CO, 2.6% for C_2_H_4_, 31.2% for CH_3_COOH, and 64.1% for H_2_, respectively. (iii) Cu_0.5_Pd_0.5_/TiO_2_ displays the selectivity of 3.7% for CO, 11.5% for H_2_, and 84.8% for CH_3_COOH, respectively. In comparison, Pd_0.5_/TiO_2_ exhibits the selectivity of 9.8% for CO and 90.2% for H_2_, respectively. The above results clearly reveal the importance of co‐loading Pd SAs and Cu SAs for obviously boosting the C─C coupling and CO_2_‐to‐CH_3_COOH conversion. The detailed roles of Pd and Cu SAs will be explored and discussed later. Additionally, Cu_0.5_Pd_0.5_/TiO_2_ exhibits an apparent quantum efficiency (AQE) of 3.28% at λ = 365 nm for CH_3_COOH evolution. Furthermore, Figure 3d exhibits the control experiment results: (i) the application of deionized (DI) water instead of seawater arouses an apparent reduction in CH_3_COOH yield to 45.4 µmol/g_cat_ and an increase in the H_2_ evolution to 405.2 µmol/g_cat_, while the CO evolution remains almost the same at 20.81 µmol/g_cat_. (ii) the absence of aluminium foil results in the reduction of CH_3_COOH yield to 51.8 µmol/g_cat_ and  reduction of H_2_ production to 241.5 µmol/g_cat_. Meanwhile, the CO production remains almost the same at 20.88 µmol/g_cat_. (iii) the application of an Argon atmosphere instead of the CO_2_ atmosphere leads to the production of H_2_ only (103.8 µmol/g_cat_). (iv) without light irradiation or catalyst presence, no products can be detected. Accordingly, the above results reveal that: (i) the seawater plays an important role on raising the CH_3_COOH and H_2_ evolution over Cu_0.5_Pd_0.5_/TiO_2_. This is because that the intrinsic properties of seawater, for example, its electrolytic content (Na^+^, Cl^−^, Mg^2^^+^, etc.), can enhance the ionic strength and solution conductivity, which can mitigate charge recombination and promote more efficient charge separation/transport across photocatalyst surface to facilitate redox reactions at the interface [51]; (ii) the application of aluminium foil, which can increase the light harvesting and reaction temperature, also serves a key role on boosting the production of CH_3_COOH over Cu_0.5_Pd_0.5_/TiO_2_; (iii) the CO_2_ gas is the carbon source for producing CH_3_COOH and CO by Cu_0.5_Pd_0.5_/TiO_2_; iv) the observed products (CH_3_COOH, H_2_, and CO) arise from the photoreduction of CO_2_ in the presence of water enabled by Cu_0.5_Pd_0.5_/TiO_2_ catalyst. Also, the above results imply the key roles of seawater and elevated reaction temperature for enhancing the C─C coupling process and improving both yield and selectivity for CH_3_COOH production over Cu_0.5_Pd_0.5_/TiO_2_.

Moreover, Figure 3e exhibits the robustness test results for Cu_0.5_Pd_0.5_/TiO_2_ over three consecutive reaction cycles (each cycle with 6‐h reaction). The first 6‐h cycle results in the production of CH_3_COOH (119.2 µmol/g_cat_), H_2_ (64.7 µmol/g_cat_), CO (20.85 µmol/g_cat_)_,_ respectively. After 12‐h light irradiation, the CO level is decreased to 10 µmol/g_cat_, while H_2_ level is increased to 93.6 µmol/g_cat_. The reduction in the generated amount of CO can be attributed to the further conversion of partial produced CO into the C_2_ products. Meanwhile, the CH_3_COOH level is further raised to 159.7 µmol/g_cat_, and C_2_H_4_ level is at 2.6 µmol/g_cat_. Further extending the light irradiation to 18 h results in an increase of CO level to 27.7 µmol/g_cat_, H_2_ level to 355 µmol/g_cat_, CH_3_COOH level to 210 µmol/g_cat_, and C_2_H_4_ level to 3.6 µmol/g_cat_. The above results clearly confirm the good robustness of Cu_0.5_Pd_0.5_/TiO_2_ for the generation of carbon‐based products from CO_2_ PR in the 18‐h reaction.

To aim for the realistic application of the as‐prepared photocatalyst, we have manufactured a floatable artificial leaf (AL) by utilizing an air spray technique to uniformly anchor the powder‐formed Cu_0.5_Pd_0.5_/TiO_2_ onto the surface of glass fiber filter paper as the substrate, as exhibited in Figure 3f. This floatable AL with porous‐structured glass fiber filter paper as the substrate can boost the interfacial contact among the CO_2_, water and catalyst at the tri‐phase interface. After 24‐h xenon light irradiation, this floatable AL have generated 31.9 µmol/g_cat_ of CO, 121.5 µmol/g_cat_ of H_2_, 3.1 µmol/g_cat_ of C_2_H_4_, and 66.4 µmol/g_cat_ of CH_3_COOH. The floatable AL exhibits selectivity of 7.3% for CO, 27.7% for H_2_, 4.3% for C_2_H_4_, and 60.7% for CH_3_COOH, respectively. Additionally, the robustness of floatable AL membrane was tested by utilizing ICP‐MS to detect the Cu and Pd leaching levels after a 24‐hour catalytic cycle in seawater. The results exhibit only 63 ppb of Cu and 105 ppb of Pd, respectively, confirming the excellent robustness of the fabricated AL. These results highlight the strong potential of Cu_0.5_Pd_0.5_/TiO_2_ catalyst for scaling up CO_2_ PR in practical application, because this stand‐alone design of AL can facilitate the deployment and recovery of powder‐formed photocatalyst, as well as accelerate the mass transfer in the CO_2_ PR process for efficient CO_2_ conversion.

Finally, we present a comparative analysis of the photocatalytic CO_2_ reduction performance over Cu_0.5_Pd_0.5_/TiO_2_ against previously reported catalysts (Figure 3g). Notably, Cu_0.5_Pd_0.5_/TiO_2_ demonstrates a remarkable balance between the yield and selectivity of CH_3_COOH from CO_2_ PR, positioning it among one of the most effective photocatalysts reported for photocatalytic CO_2_‐to‐CH_3_COOH conversion. Moreover, key reaction parameters and performance metrics are detailed in Table S3. Although the reported Pd‐Co_3_O_4_ exhibits a 100% selectivity for CH_3_COOH evolution, the additional energy input is required to keep the whole reaction system at 60°C and Pd‐Co_3_O_4_ also presents an apparently lower rate for CH_3_COOH evolution (13.8 µmol g^−1^ h^−1^; Table S3), compared to our work (19.8 µmol g^−1^ h^−1^). And no external energy input is required in our work. Furthermore, although the reported NiCo‐TiO_2_ exhibits a higher rate for CH_3_COOH evolution (22.6 µmol g^−1^ h^−1^; Table S3), compared to Cu_0.5_Pd_0.5_/TiO_2_ (19.8 µmol g^−1^ h^−1^), sacrificial agent (Na_2_SO_3_) and alkaline chemical (CsOH) are added in the reaction system (Table S3). In contrast, no sacrificial agent or alkaline chemical is added in our work. Therefore, we can convincingly highlight that our catalyst Cu_0.5_Pd_0.5_/TiO_2_ ranks among one of the most efficient catalysts for photocatalytic CO_2_ reduction to CH_3_COOH in the absence of additional energy input, sacrificial agent, or alkaline trapping chemical. This enhanced photocatalytic activity is attributed to the synergistic effects arising from the dual incorporation of Cu and Pd SAs sites onto the TiO_2_ surface, which significantly improves charge separation and facilitates the selective reduction of CO_2_ to CH_3_COOH. For the oxidation half reaction, we have tried to utilize a UV‐Vis spectrophotometric technique to detect the H_2_O_2_ evolution and gas chromatography equipped with a thermal conductivity detector (GC‐TCD) to detect the O_2_ evolution, respectively. Unfortunately, under the explored reaction condition, neither H_2_O_2_ nor O_2_ was detected within the detection limits of the respective analytical techniques. These results reveal that stable oxidation products of H_2_O_2_/O_2_ don't accumulate to measurable concentration in the reaction process. Although neither O_2_ nor H_2_O_2_ was detected under the explored reaction conditions, the absence of these products does not necessarily exclude the occurrence of oxidation half‐reactions. For example, photo‐generated holes can generate highly reactive oxygen species, for example, hydroxyl radical (^*^OH), which possess extremely short lifetimes and can be rapidly consumed in interfacial reactions (^*^CH_3_CO + ^*^OH → ^*^CH_3_COOH) before being accumulated to detectable concentrations. Furthermore, seawater contains a range of dissolved inorganic ions (e.g., Cl^−^ and Br^−^) that could also undergo hole‐driven oxidation. Therefore, oxidation equivalents may be distributed among multiple competing pathways rather than accumulating as measurable O_2_ or H_2_O_2_. Consequently, the lack of detectable O_2_ or H_2_O_2_ cannot be interpreted as evidence for the absence of oxidation processes in this reaction system. Actually, the absence of detectable oxidation products are also extensively reported in previous references (Table S3).

To identify the carbon source of generated products, ^13^CO_2_ isotope‐labeling experiments were conducted and corresponding results are exhibited in Figures 3h,i and S13a,b. As exhibited in Figure 3h, the mass spectrum of produced ^13^CH_3_^13^COOH exhibits characteristic signals at m/z = 62, 47, 46, 45, 44, 32, 30, and 16, which can be assigned to ^13^CH_3_^13^COOH^+^, ^13^COOH^+^, ^13^C_2_H_4_O^+^, ^13^CH_3_^13^CO^+^, ^13^C_2_H_2_O^+^, H_2_^13^COH^+^, H^13^CO^+^, and ^13^CH_3_^+^ fragments, respectively. Notably, the appearance of the molecular ion peak at m/z = 62 corresponding to ^13^CH_3_^13^COOH unambiguously confirms that both carbon atoms in the generated acetic acid originate from ^13^CO_2_. In addition, the mass spectrum of CO exhibits a dominant ^13^CO signal (m/z = 29) with only a negligible contribution from ^12^CO (m/z = 28), yielding a ^13^CO/^12^CO ratio of ∼99:1 (Figure 3i) [85].

### 2.3 Charge Carrier Kinetics

First, the optical characteristics and electronic band structure of the as‐prepared photocatalysts have been thoroughly investigated to elucidate the key factor resulting in their enhanced photocatalytic efficacy. The UV‐Vis diffuse reflectance spectra of the as‐prepared catalysts (Figure S14) present that the incorporation of Cu SAs onto the TiO_2_ matrix raises the light absorption capacity of Cu_0.5_/TiO_2_ in the range of ∼200–800 nm, compared to pristine TiO_2_. Furthermore, the addition of Pd SAs onto Cu_0.5_/TiO_2_ catalyst further broadens the light absorption spectrum of the optimized Cu_0.5_Pd_0.5_/TiO_2_ catalyst. Accordingly, the calculated optical bandgap energies (Eg) for TiO_2_, Cu_0.5_/TiO_2_, Cu_0.5_Pd_0.5_/TiO_2_, and Pd_0.5_/TiO_2_ are 3.08, 2.93, 2.82, and 2.82 eV, respectively. The corresponding change of colors are also reflected by the photographs of TiO_2_, Cu_0.5_/TiO_2_, and Cu_0.5_Pd_0.5_/TiO_2_ (Figure S14 inset). Furthermore, the Mott‐Schottky (MS) analyses (Figure S15) reveal the electronic band structures of TiO_2_ and Cu_0.5_Pd_0.5_/TiO_2_: (i) First, the flat band potentials are determined to be −0.51 and −0.49 V vs. the Ag/AgCl electrode for TiO_2_ and Cu_0.5_Pd_0.5_/TiO_2_, respectively. (ii) Thus, the Fermi level (EF) of TiO_2_ and Cu_0.5_Pd_0.5_/TiO_2_ are estimated to be at 0.12 and 0.14 V vs. the reversible hydrogen electrode (RHE), respectively. iii) Consequently, the valence band (VB) edge positions of TiO_2_ and Cu_0.5_Pd_0.5_/TiO_2_ are calculated to be at 2.97 and 2.14 V vs. RHE, respectively, based on the XPS VB spectra (Figure S16). iv) As a result, the CB edge positions of TiO_2_ and Cu_0.5_Pd_0.5_/TiO_2_ are determined to be at −0.11 and −0.68 V vs. RHE, respectively. The above calculated results on the electronic band structures, as exhibited in Figure S17, clearly reveal the movement of the CB edge position to the much higher position for Cu_0.5_Pd_0.5_/TiO_2_, compared to that of TiO_2_. This movement highlights the greatly enhanced electron donation/injection ability and catalytic reduction capacity of Cu_0.5_Pd_0.5_/TiO_2_, compared to bare TiO_2_, which is crucial for the formation of C_2_ products.

**FIGURE 4:**
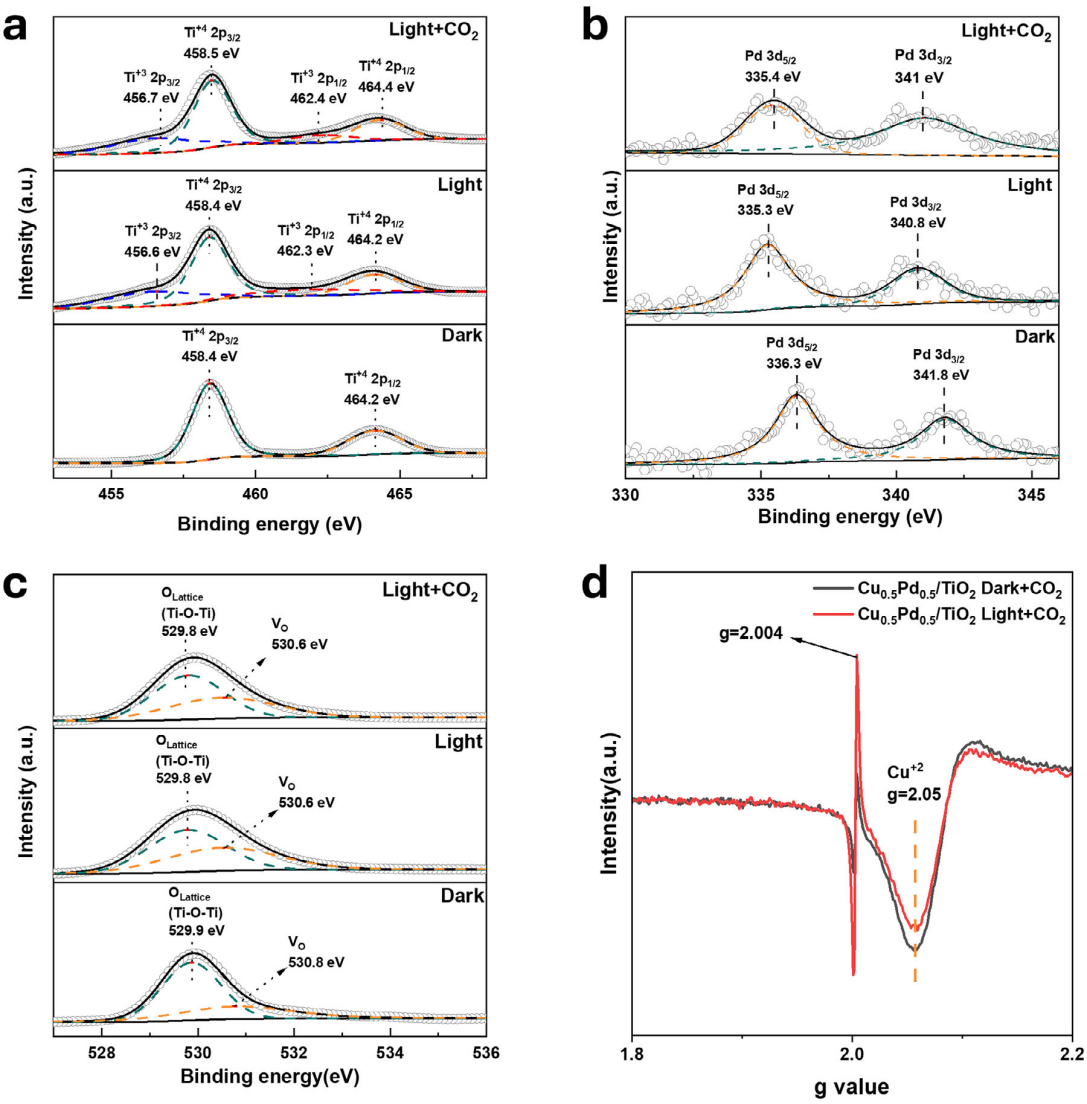
In situ high resolution XPS spectra of (a) Ti 2p, (b) Pd 3d and (c) O 1s peaks for Cu_0.5_Pd_0.5_/TiO_2_ photocatalyst under the following three different conditions: (i) in the dark and vacuum condition; (ii) under light irradiation and in vacuum condition; and (iii) under light irradiation and in CO_2_ atmosphere (light + CO_2_), respectively. (d) In situ EPR spectra for Cu_0.5_Pd_0.5_/TiO_2_ photocatalyst in dark condition and CO_2_ atmosphere (dark + CO_2_), as well as under light irradiation and in CO_2_ atmosphere (light + CO_2_), respectively.

Moreover, the steady‐state photoluminescence (PL) spectra results (Figure S18) presents an apparent reduction in the PL intensity of Cu_0.5_Pd_0.5_/TiO_2_, compared to those of TiO_2_, Cu_0.5_/TiO_2_ or Pd_0.5_/TiO_2_ [72] These results clearly confirm the decreased recombination rates and enhanced carrier separation efficiency of the optimized Cu_0.5_Pd_0.5_/TiO_2_, compared to those of the other catalysts, which result in the most active performance for Cu_0.5_Pd_0.5_/TiO_2_. To further comprehensively reveal the charge‐transfer kinetics between Cu/Pd SAs and TiO_2_, in situ XPS and in situ EPR techniques were employed. In dark and in vacuum condition, the high‐resolution Ti 2p spectrum of Cu_0.5_Pd_0.5_/TiO_2_ (Figure 4a) exhibit two peaks located at 458.4 and 464.2 eV, attributed to the Ti 2p_3/2_ and Ti 2p_1/2_ peaks for Ti^4+^. Under light irradiation and in vacuum condition, Figure 4a exhibit two new peaks located at 456.6 and 462.3 eV, attributed to the Ti 2p_3/2_ and Ti 2p_1/2_ peaks for Ti^3+^. These results reveal that with light irradiation, the photo‐generated electrons reduce partial Ti^4+^, resulting in the formation of Ti^3+^. Nevertheless, the positions of Ti 2p_3/2_ (458.4 eV) and Ti 2p_1/2_ (464.2 eV) peaks for Ti^4+^ of Cu_0.5_Pd_0.5_/TiO_2_ remains unchanged under light irradiation and in vacuum condition, compared to those in dark and in vacuum condition. These results indicate that most of the Ti^4+^ of Cu_0.5_Pd_0.5_/TiO_2_ doesn't capture the photo‐generated electrons. Furthermore, under light irradiation and in CO_2_ atmosphere, all the four Ti 2p peaks of Cu_0.5_Pd_0.5_/TiO_2_ (Figure 4a) are moved towards the higher binding energy direction by 0.1/0.2 eV, compared to those under light irradiation and in vacuum condition. These results clearly reveal that the purged CO_2_ molecules are adsorbed on the surface of Cu_0.5_Pd_0.5_/TiO_2_ and extract the electrons from Ti^3+^/Ti^4+^ of Cu_0.5_Pd_0.5_/TiO_2_. Moreover, Figure 4b exhibits the presence of Pd 3d_5/2_ and Pd 3d_3/2_ peaks located at 336.3 and 341.8 eV for Cu_0.5_Pd_0.5_/TiO_2_ in dark and vacuum condition. Interestingly, under light irradiation and in vacuum condition, the Pd 3d_5/2_ and Pd 3d_3/2_ peaks of Cu_0.5_Pd_0.5_/TiO_2_ (Figure 4b) are apparently moved to the lower binding energy direction by 1.0 eV. These results unambiguously indicate that the anchored Pd SAs strongly extract the photo‐generated electrons from the TiO_2_ support in Cu_0.5_Pd_0.5_/TiO_2_ with light excitation. Furthermore, under light irradiation and in CO_2_ atmosphere, the Pd 3d_5/2_ and Pd 3d_3/2_ peaks of Cu_0.5_Pd_0.5_/TiO_2_ only exhibit a slight movement towards the higher binding energy direction by 0.1 eV. These results unveil that most of the photo‐generated electrons captured by Pd SAs are not transferred to CO_2_ molecules adsorbed on the surface of Cu_0.5_Pd_0.5_/TiO_2_ but are still trapped by the Pd SAs.

These results also imply that Pd SAs of Cu_0.5_Pd_0.5_/TiO_2_ are not ideal adsorption and reduction sites for CO_2_ molecules. Furthermore, in dark and vacuum condition, the deconvoluted spectrum of O 1s peaks for Cu_0.5_Pd_0.5_/TiO_2_ (Figure 4c) exhibit one main peak at 529.9 eV and one small peak at 530.8 eV, attributed to the lattice O with Ti─O─Ti bonding and O vacancy (VO) in TiO_2_ of Cu_0.5_Pd_0.5_/TiO_2_ catalyst. Under light irradiation and in vacuum condition, these two peaks are moved towards the lower binding energy direction by 0.1/0.2 eV (Figure 4c). These results indicate that some photo‐excited electrons are captured by lattice O atoms and V_O_ with light excitation. Additionally, with light irradiation and in CO_2_ atmosphere, no perceivable change of peak position is found for these two O 1s peaks of Cu_0.5_Pd_0.5_/TiO_2_ (Figure 4c), compared to those under light irradiation and in vacuum condition.

These results indicate that either lattice O atoms or V_O_ contribute any electrons for CO_2_ reduction, probably owing to their high electron negativity and strong electron‐trapping capacity. In contrast, as exhibited in Figure S19a, the Ti 2p spectra of bare TiO_2_ exhibit two peaks located at 458.4 and 464.2 eV, corresponding to the Ti 2p_3/2_ and Ti 2p_1/2_ peaks for Ti^4+^, respectively, in dark and vacuum conditions. In comparison, under light irradiation, the Ti 2p spectra of TiO_2_ alone exhibit two new peaks located at 456.5 and 462.1 eV (Figure S19a), respectively, attributed to the Ti 2p_3/2_ and Ti 2p_1/2_ peaks for Ti^3+^. These results also indicate that the photo‐excited electrons reduce partial Ti^4+^ to generate Ti^3+^. Moreover, under light irradiation and in CO_2_ atmosphere, the two Ti 2p peaks related to Ti^3+^ for TiO_2_ exhibit the apparent movement towards the higher binding energy direction by 0.3 eV, more obvious than the Ti 2p peak movements (0.1 eV) for Cu_0.5_Pd_0.5_/TiO_2_ under the identical conditions (Figure 4a). These results indicate that for pure TiO_2_, more CO_2_ molecules are adsorbed near/on the Ti^3+^ sites, followed by the electron transfer from Ti^3+^ sites to these adsorbed CO_2_ molecules under light irradiation. These results also imply that less CO_2_ molecules are adsorbed near/on Ti^3+^ sites of Cu_0.5_Pd_0.5_/TiO_2_ but near/on the Pd/Cu SAs. Furthermore, in dark and vacuum condition, Figure S19b exhibits two O 1s peaks located at 529.9 and 531.3 eV, corresponding to the Ti‐O‐Ti (lattice O) and V_O_ of pure TiO_2_, respectively. Interestingly, with light irradiation and in vacuum condition, the O 1s peak related to V_O_ in pure TiO_2_ (Figure S19b) exhibits the apparent movement towards the lower binding energy direction by 0.4 eV, more obvious than the O 1s peak movement for Cu_0.5_Pd_0.5_/TiO_2_ under the identical conditions (Figure 4c). These results reveal that without Pd/Cu SAs loading, more photo‐generated electrons are captured by V_O_ in TiO_2_. Additionally, in CO_2_ atmosphere, the in situ EPR test results with/without light irradiation were collected for bare TiO_2_ (Figure S20) and Cu_0.5_Pd_0.5_/TiO_2_ (Figure 4d), respectively. In CO_2_ atmosphere and without light irradiation, pure TiO_2_ only exhibits a weak resonance signal at g = 2.004 (Figure S20), attributed to the presence of a small amount of V_O_ in TiO_2_. In comparison, in CO_2_ atmosphere and under light irradiation, pure TiO_2_ presents the increased resonance signal at g = 2.004, with an intensity about ∼2 times of that in the dark condition. These results reveal the in situ production of V_O_ in TiO_2_ after light excitation, arising from the oxidation of O_2_^−^ in TiO_2_ by photo‐excited holes [86]. For Cu_0.5_Pd_0.5_/TiO_2_, without light irradiation and in CO_2_ atmosphere, Cu_0.5_Pd_0.5_/TiO_2_ exhibits a more intense resonance signal at g = 2.004 than TiO_2_. This indicates the presence of more V_O_ in Cu_0.5_Pd_0.5_/TiO_2_ than that in TiO_2_, probably generated in the process for sequential loading of dual SAs Cu and Pd on TiO_2_. The resonance signal centered at g ≈ 2.05 is characteristic of paramagnetic Cu^2^^+^ species possessing a 3d^9^ electronic configuration, while Cu^+^ (3d^10^) and Cu^0^ are generally EPR silent because of their diamagnetic electronic structures. The assignment of Cu^2+^ peak in Figure 4d is extensively corroborated by abundant previous references [72, 87]. Notably, this assignment is also corroborated by the Cu K‐edge XANES results, in which the absorption edge position is located close to that of the CuO reference, indicating a predominantly oxidized Cu^2+^ state. Furthermore, EXAFS and WT‐EXAFS analyses reveal isolated Cu‐O coordination without detectable Cu─Cu metallic bonding. Therefore, the combined evidence from XANES, EXAFS/WT‐EXAFS, and EPR consistently corroborates the presence of atomically dispersed oxidized Cu species in Cu_0.5_Pd_0.5_/TiO_2_. Under light irradiation in a CO_2_ atmosphere, Cu_0.5_Pd_0.5_/TiO_2_ exhibits an apparently enhanced oxygen‐vacancy‐related EPR signal compared with pristine TiO_2_, revealing more efficient charge separation and charge accumulation at vacancy‐related sites. Meanwhile, the Cu^2^^+^‐related resonance at g ≈ 2.05 exhibits a moderate decrease in intensity under light irradiation, indicating partial electron transfer from the photoexcited TiO_2_ to Cu centers. This observation is consistent with the role of Cu sites as electron‐accepting centers in photocatalysis.

Through combining the above in situ EPR results (Figure 4d) and in situ XPS results of Cu_0.5_Pd_0.5_/TiO_2_ (Figure 4a–c), we can acquire some insights into the photo‐excited electron and hole separation/transfer mechanism for Cu_0.5_Pd_0.5_/TiO_2_ under light irradiation and in CO_2_ atmosphere: i) Pd SAs can strongly extract the photo‐excited electrons from TiO_2_ in Cu_0.5_Pd_0.5_/TiO_2_ and these Pd SAs can retain most of the captured electrons even when CO_2_ molecules are adsorbed on the surface. ii) Most of the Cu SAs are with an average valence status of +2 in Cu_0.5_Pd_0.5_/TiO_2_. These Cu SAs can also attract photo‐excited electrons from TiO_2_, even though not that efficient compared to Pd SAs. iii) Considering both Pd and Cu SAs pumping plentiful photo‐excited electrons from TiO_2_, much more photo‐generated holes can remain in TiO_2_ and thus oxidize ^·^O_2_^−^ to in situ yield abundant V_O_ in TiO_2_ of Cu_0.5_Pd_0.5_/TiO_2_. iv) Apart from Pd and Cu SAs, V_O_ of Cu_0.5_Pd_0.5_/TiO_2_ can also extract photo‐excited electrons from the CB of TiO_2_, but less effective in Cu_0.5_Pd_0.5_/TiO_2_ than V_O_ in TiO_2_. This is because a major part of photo‐excited electrons is captured by Pd/Cu SAs on the surface of Cu_0.5_Pd_0.5_/TiO_2_.

**FIGURE 5:**
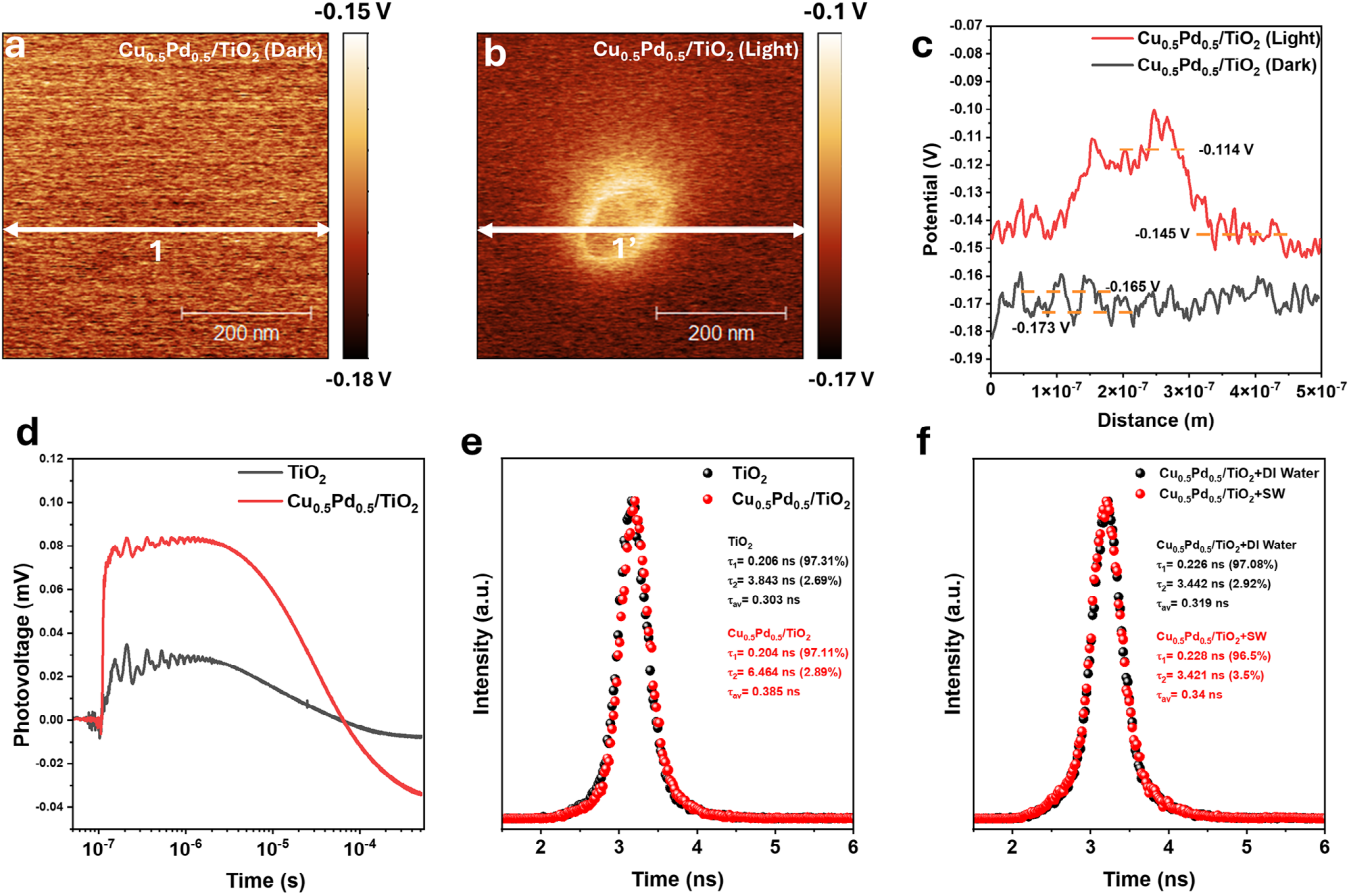
(a) KPFM image of Cu_0.5_Pd_0.5_/TiO_2_ in dark condition and in air atmosphere. (b) KPFM image of Cu_0.5_Pd_0.5_/TiO_2_ with light irradiation and in air atmosphere. (c) The corresponding potential profiles of Cu_0.5_Pd_0.5_/TiO_2_ along line 1 and line 1’ in dark and under light irradiation, respectively. (d) In situ transient‐state SPV spectra of TiO_2_ and Cu_0.5_Pd_0.5_/TiO_2_ in air atmosphere. (e) TP spectra of TiO_2_ and Cu_0.5_Pd_0.5_/TiO_2_. (f) In situ TP of Cu_0.5_Pd_0.5_/TiO_2_ in DI water and SW, respectively.

Furthermore, in situ AFM‐KPFM measurements were performed to analyze the contact potential difference (CPD) between the tip work function (Φ_tip_) and sample work function (Φ_sample_), which can indirectly reflect the nanometre‐level spatial distribution of photo‐excited charge carriers on the surface of catalyst under light irradiation and in certain atmosphere, for example, air. As exhibited in Figure S21a–d, the AFM images (Figure S21a,c) and the corresponding line‐analyze information (Figure S21b,d) indicate no apparent topography change (e.g., height and size) for Cu_0.5_Pd_0.5_/TiO_2_, after changing the test condition from in darkness (Figure S21a–b) to with light irradiation (Figure S21c,d). Actually, the AFM images in Figure S21a,c clearly reveal the typical topography of aggregated TiO_2_ nanoparticles (P25 TiO_2_) with heights of ∼39 nm for Cu_0.5_Pd_0.5_/TiO_2_. The same region was further explored, and the corresponding surface potential maps and line scans (1 and 1’) in dark conditions or under light irradiation are exhibited in Figure 5a–c. As exhibited in Figure 5c, in dark condition and air atmosphere, the surface potential difference between the base and the aggregated nanoparticles of Cu_0.5_Pd_0.5_/TiO_2_ is almost negligible, revealing no perceivable potential difference between the base and the surface of these aggregated nanoparticles (Cu_0.5_Pd_0.5_/TiO_2_). In contrast, with light irradiation and in air atmosphere, the surface potential difference between the base and the surface of aggregated Cu_0.5_Pd_0.5_/TiO_2_ nanoparticles are ∼41 mV, with the increased surface potential for Cu_0.5_Pd_0.5_/TiO_2_. Interestingly, the above results are obviously contradictory to the above in situ XPS results (Figure 4a–c), which present the accumulated photo‐excited electrons on the surface of Cu_0.5_Pd_0.5_/TiO_2_ (Ti, O and Pd elements) under light irradiation and in vacuum condition. This inconsistency is attributed to the presence of O_2_ molecules in air, which are adsorbed on the catalyst surface and act as the electron scavenger to effectively extract photo‐generated electrons, resulting in an excess of holes on the surface [88]. Additionally, we employed transient‐state surface photovoltage (TPV) spectroscopy to further explore the dissociation, transfer, trapping, and recombination kinetics of photo‐generated charge carriers on the surfaces of bare TiO_2_ and Cu_0.5_Pd_0.5_/TiO_2_. As exhibited in Figure 5d, after light excitation by a 355 nm laser, TiO_2_ presents the gradual increase of the positive photovoltage signal in the time range of ∼110 ns to ∼150 ns, reaching the highest positive photovoltage signal of ∼0.033 mv at ∼211 ns. In comparison, Cu_0.5_Pd_0.5_/TiO_2_ presents a rapid increase of positive photovoltage signal starting from ∼106 ns, reaching ∼0.069 mv at ∼121 ns. Then, Cu_0.5_Pd_0.5_/TiO_2_ exhibits a gradual enhancement of the positive photovoltage signal from ∼133 to ∼155 ns, reaching the highest positive signal of ∼0.082 mv at ∼211 ns. Through comparing the above TPV results of TiO_2_ and Cu_0.5_Pd_0.5_/TiO_2_, we can observe the following two phenomenon: (i) Compared to pure TiO_2_, Cu_0.5_Pd_0.5_/TiO_2_ possesses a much quicker increasing rate of positive photovoltage signal and (ii) Cu_0.5_Pd_0.5_/TiO_2_ reaches a much higher maximum positive signal (0.082 mv), in contrast with TiO_2_ alone (0.0335 mv). Both TiO_2_ and Cu_0.5_Pd_0.5_/TiO_2_ first exhibit the positive signal owing to the following two reasons: (i) after the light excitation, many of the photo‐generated electrons are captured by the oxygen vacancies (V_O_) in the bulk of TiO_2_ or Cu_0.5_Pd_0.5_/TiO_2_; while the remaining photo‐generated holes are separated and transported to the surface of catalyst. (ii) when both photo‐generated electrons and holes migrate to the surface of TiO_2_ or Cu_0.5_Pd_0.5_/TiO_2_, many of the photo‐generated electrons are captured by the adsorbed O_2_ molecules from the air (O_2_ + e^−^ = ^∙^O_2_^−^), leaving the holes remaining on the surface of catalyst. Moreover, as exhibited in Figure 5d, compared to TiO_2_, Cu_0.5_Pd_0.5_/TiO_2_ not only demonstrates a much quicker raise of positive photovoltage signal, but also presents an apparently higher positive photovoltage signal. These results are attributed to the following two reasons: (i) in contrast with TiO_2_, Cu_0.5_Pd_0.5_/TiO_2_ presents a higher content of V_O_ in the bulk, resulting in more trapped electrons and more separated holes migrating to the surface; (ii) the presence of both Cu SAs and Pd SAs on the surface of TiO_2_ results in the extraction and accumulation of photo‐induced electrons at the Cu/Pd SAs only, rather than the abundant surface of TiO_2_. Thus, for testing the whole surface area of TiO_2_ or Cu_0.5_Pd_0.5_/TiO_2_ by TPV technique (Figure 5d), Cu_0.5_Pd_0.5_/TiO_2_ exhibits a much stronger positive signal than TiO_2_ for the overall surface of catalyst. Then, both TiO_2_ and Cu_0.5_Pd_0.5_/TiO_2_ present the gradually reducing positive photovoltage signal from their highest positive signals to zero, further to the maximum negative signal at ∼5.188 x 10^−4^ s. The above results are attributed to the following reasons: i) the remaining holes can be gradually consumed by the surface adsorbed ‐OH (h^+^ + OH^−^ = ^∙^OH) and the generated ^∙^O_2_^−^ (^∙^O_2_^−^ + h^+^ = ^1^O_2_). ii) many electrons temporarily trapped at the shallow donor levels of V_O_ are gradually released and migrate from the bulk to the surface of TiO_2_ or Cu_0.5_Pd_0.5_/TiO_2_, resulting in an increasing negative potential. Notably, Cu_0.5_Pd_0.5_/TiO_2_ exhibits a slower SPV decay compared to pure TiO_2_ (Figure 5d), indicating that the incorporation of Pd and Cu SAs facilitates enhanced charge separation and transfer. These above results align with the in situ EPR results (Figures 4d and S20) and in situ XPS findings (Figure 4a–c). All these results demonstrate that adding dual single atoms onto TiO_2_ greatly improves the lifetime of photogenerated carriers on its surface. To further assess the efficiency of charge carrier separation, we conducted TP measurements on bare TiO_2_ and Cu_0.5_Pd_0.5_/TiO_2_. As illustrated in Figure 5e, the TP decay spectra reveal the slower photoluminescence decay for Cu_0.5_Pd_0.5_/TiO_2_ compared to that for TiO_2_, with average charge lifetimes (τ_ave_) of 0.385 ns (Cu_0.5_Pd_0.5_/TiO_2_) and 0.303 ns (TiO_2_), respectively. The longer averaged charge lifetime indicates that the incorporation of Pd and Cu SAs significantly inhibits charge recombination, thereby enhancing the charge separation efficiency in Cu_0.5_Pd_0.5_/TiO_2_. Finally, to elaborate on the pivotal role of seawater in accelerating the charge dynamics of Cu_0.5_Pd_0.5_/TiO_2_, in situ TP experiments were conducted in both deionized water and seawater environments (Figure 5f). The analysis of the TP decay spectra for Cu_0.5_Pd_0.5_/TiO_2_ reveals two distinct decay components, as detailed in the inset of Figure 5f. The short component, associated with intrinsic exciton recombination in the bulk of Cu_0.5_Pd_0.5_/TiO_2_ [89], remained relatively unaffected by the solvent, with 0.218 ns (τ_1_) in deionized water and 0.228 ns (τ_2_) in seawater. This stability indicates that the local electric field generated by the abundant cations and anions in seawater predominantly impacts surface charge separation and migration rather than the bulk region. In contrast, the longer decay component features significant reliance on the solution conditions, with τ_2_ of 3.104 ns (3.66%) in deionized water and τ_2_ of 3.686 ns (3.38%) in seawater [51]. This change is attributed to enhanced electron‐hole recombination suppression at the surface of Cu_0.5_Pd_0.5_/TiO_2_, facilitated by the local electric fields from the ionic environment in seawater. Consequently, the average charge lifetime for Cu_0.5_Pd_0.5_/TiO_2_ is increased from τ_ave_ of 0.323 ns in deionized water to τ_ave_ of 0.345 ns in seawater (Figure 5f). Notably, a parallel trend is also observed in both TP results for TiO_2_ and Cu_0.5_Pd_0.5_/TiO_2_ (Figure 5e), of which the longer component for TiO_2_ (τ_2_ = 3.843 ns; 2.69%) compared to that for Cu_0.5_Pd_0.5_/TiO_2_ (τ_2_ = 6.464 ns; 2.89%).

### 2.4 Reaction Mechanism

**FIGURE 6:**
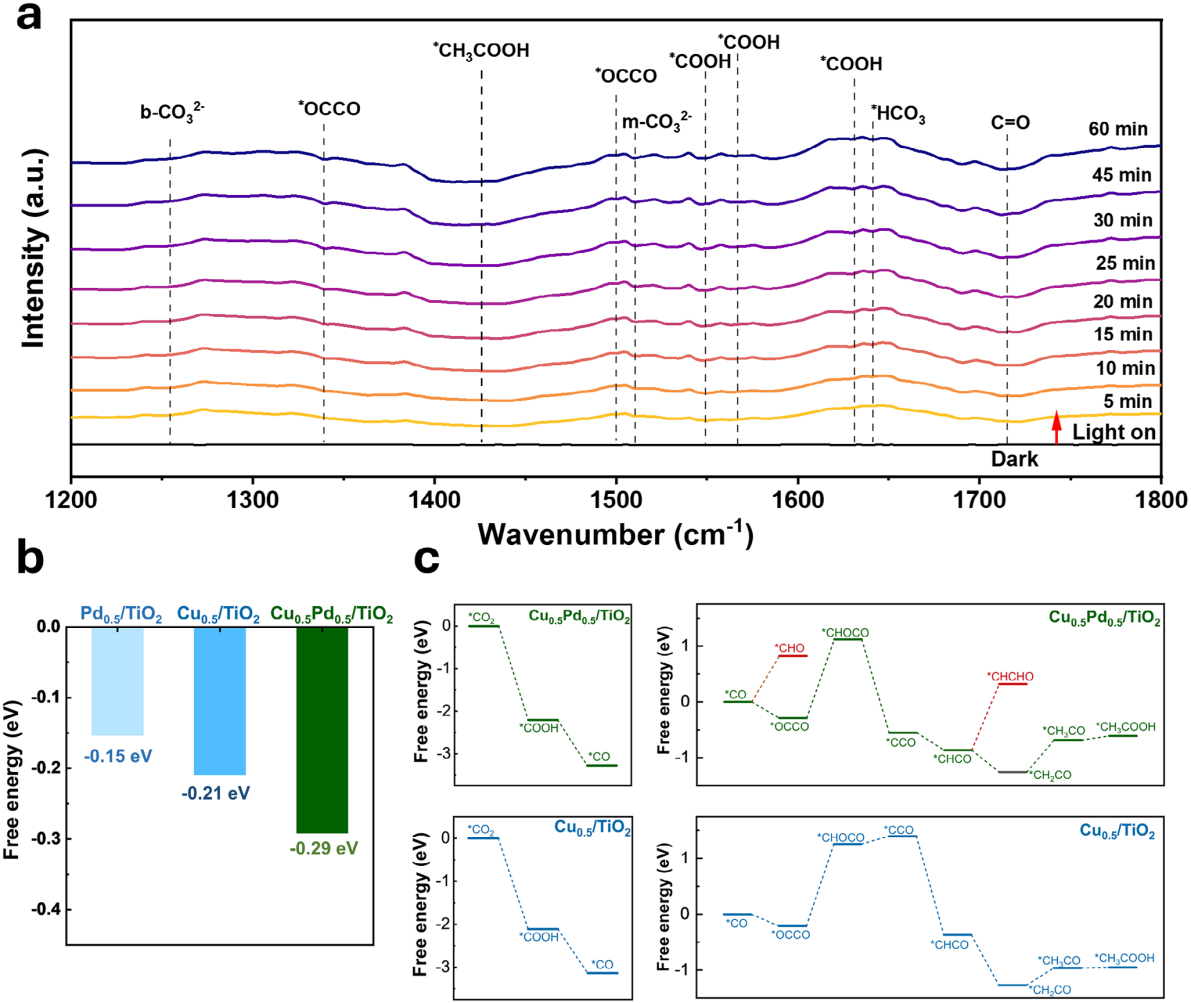
(a) In situ IR spectra for the CO_2_ PR over Cu_0.5_Pd_0.5_/TiO_2_ in the presence of CO_2_ and at a temperature of 65°C. (b) The calculated Gibbs free energy changes (ΔG) for the C─C coupling step over Pd_0.5_/TiO_2_, Cu_0.5_/TiO_2_, and Cu_0.5_Pd_0.5_/TiO_2_ models, respectively. (c) The calculated Gibbs free energy diagrams for CO_2_‐to‐CH_3_COOH conversion via C─C coupling over Cu_0.5_/TiO_2_ and Cu_0.5_Pd_0.5_/TiO_2_ models, respectively.

Both the optoelectronic characteristics and charge carrier dynamics demonstrate that the Cu_0.5_Pd_0.5_/TiO_2_ catalyst favors more efficient photo‐generated electron distribution under light irradiation in a CO_2_‐rich environment. As a result, we investigated the reasons for the increased yields and selectivity for CH_3_COOH in the Cu_0.5_Pd_0.5_/TiO_2_ system. This was supported by experimental findings and theoretical analyses. To uncover the catalytic reaction mechanism for the CO_2_ reduction reaction, we employed in situ IR spectroscopy to track key reaction intermediates over time. As depicted in Figure 6a, a range of peaks are intensified with increasing light irradiation time in CO_2_ atmosphere and at an elevated temperature of 65°C. These conditions simulate the real reaction environments. The peaks at 1254 cm^−1^ and 1510 cm^−1^ correspond to the stretching vibrations of bidentate (b‐CO_3_^2−^) and monodentate (m‐CO_3_^2−^) carbonates, respectively [31]. Notably, the asymmetric stretching of bicarbonate ^*^HCO_3_ is observed at 1642 cm^−1^ [80]. Furthermore, signals at 1548 cm^−1^, 1567 cm^−1^, and 1631 cm^−1^ are attributed to the formation of ^*^COOH species, which serve as critical intermediates in the conversion of CO_2_ to carbon monoxide (CO) and subsequent C_2_ hydrocarbons [38, 78]. Key intermediates attributed to CH_3_COOH synthesis, represented as ^*^OCCO, are identified by bands at 1340 cm^−1^ and 1499 cm^−1^, indicating the potential for C─C coupling pathways on the Cu_0.5_Pd_0.5_/TiO_2_ surface [31, 78]. Additionally, the C = O stretching peak for CH_3_COOH is detected at 1715 cm^−1^ [51], while the peak at 1430 cm^−1^ serves as direct evidence for CH_3_COOH production [31, 78, 80], confirming the effectiveness of the catalyst (Cu_0.5_Pd_0.5_/TiO_2_) in facilitating the desired reaction pathways. In comparison, in situ IR spectra for Cu_0.5_/TiO_2_ (Figure S22a) and Pd_0.5_/TiO_2_ (Figure S22b) only exhibit signals at 1240 and 1575 cm^−1^, attributed to the formation of b‐CO_3_^2−^. Additionally, the peaks at 1325 and 1490 cm^−1^ correspond to m‐CO_3_^2−^, and the signal at 1640 cm^−1^ is attributed to the formation of ^*^COOH species [90, 91]. These intermediates are essential in the conversion of CO_2_ to CO. Nevertheless, no apparent signals related to C_2_ oxygenates are detected on either Cu_0.5_/TiO_2_ or Pd_0.5_/TiO_2_, revealing that the subsequent C─C coupling process is kinetically unfavorable on these two catalysts. In comparison, Cu_0.5_Pd_0.5_/TiO_2_ exhibits additional surface intermediates related to deeper CO_2_ reduction and C─C bond formation (Figure 6a), highlighting the cooperative role of neighboring Cu and Pd centers in facilitating multi‐carbon product formation.

Additionally, the DFT‐based calculations further elucidate the role of Pd loading in the Cu_0.5_Pd_0.5_/TiO_2_ matrix for enhancing CO_2_ PR activity and steering the reaction selectivity toward CH_3_COOH formation. We have constructed three different configurations of active‐site structures: the first is Cu_0.5_/TiO_2_, and the second Pd_0.5_/TiO_2_, and the third is Cu_0.5_Pd_0.5_/TiO_2_ (Figure S23) exhibiting the consistent coordination environments as experimental characterizations. The adsorption and activation of CO_2_ molecules are critical beginning points for the subsequent proton‐electron transfer process. The results in Figure 6b reveal the less favorable energetics for C─C coupling over Cu_0.5_/TiO_2_ or Pd_0.5_/TiO_2_ models, compared to that over Cu_0.5_Pd_0.5_/TiO_2_ model. These indicate that the Cu‐Pd dual‐single‐atom configuration can boost ^*^OCCO formation and promote the subsequent C─C coupling process. Notably, the superior CO_2_‐to‐CH_3_COOH conversion performance over Cu_0.5_Pd_0.5_/TiO_2_ cannot be attributed to a single elementary step. Instead, it arises from the cooperative regulation of multiple key intermediates (e.g., ^*^OCCO and ^*^CCO) throughout the reaction pathway (Figure 6c). In alignment with in situ IR results, the theoretical calculation results consistently indicate that neighboring Cu and Pd sites cooperatively boost C─C coupling and thereby favor the formation of C_2_ products. Furthermore, compared with the coupling of ^*^CO species to form ^*^OCCO, the alternative pathway involving ^*^CO to ^*^CHO exhibits a much higher energy barrier of 0.82 eV (Figure 6c), indicating a thermodynamically less favorable route for the formation of other C_1_ products (e.g., CH_4_ or CH_3_OH). These results highlight the role of Pd in promoting C─C bond formation and favoring the generation of C_2_ products. Among the possible C_2_ pathways, the formation of ^*^CHCHO intermediates is found to be energetically unfavorable, with a high energy barrier of 1.18 eV on Cu_0.5_Pd_0.5_/TiO_2_, thereby limiting the production of C_2_H_4_. In comparison, the reaction pathway toward CH_3_COOH formation is thermodynamically more favorable (Figures 6c and S24), aligning well with the experimentally observed high selectivity for CH_3_COOH over Cu_0.5_Pd_0.5_/TiO_2_. Although the afore‐mentioned experimental results cannot directly reveal the function of Cu atom in C_2_ formation, the collective evidence from photocatalytic performances (Figure 3a–c), in situ IR spectra (Figures 6a and S22a,b), theoretical calculations (Figures 6b,c and S24) and established mechanistic understanding from reported references (Table S4) corroborate the proposed reaction mechanism (Figure 1) as a reliable reaction model. Notably, the theoretical calculation results (Figure 6b) are acquired to evaluate the thermodynamic feasibility of the key C─C coupling step (^*^CO + ^*^CO → ^*^OCCO) over various catalyst models. As exhibited in Figure 6b, the formation of ^*^OCCO intermediate is thermodynamically favorable on all three catalysts, with calculated free‐energy changes of −0.15, −0.21, and −0.29 eV for Pd_0.5_/TiO_2_, Cu_0.5_/TiO_2_, and Cu_0.5_Pd_0.5_/TiO_2_, respectively. Notably, the Cu_0.5_Pd_0.5_/TiO_2_ model exhibits the most negative free‐energy change (−0.29 eV), indicating the strongest stabilization of the C─C coupled intermediate (^*^OCCO). This result clearly reveals that the cooperative interaction between neighboring Cu and Pd atoms results in a more favorable reaction environment for C─C bond formation, thereby facilitating generation of C_2_ products. In contrast, Pd_0.5_/TiO_2_ model exhibits the least favorable energetics for ^*^OCCO formation (−0.15 eV); while Cu_0.5_/TiO_2_ presents more favorable energetics for ^*^OCCO formation (−0.21 eV) than Pd_0.5_/TiO_2_. Importantly, the addition of neighboring Cu atom within the Pd_0.5_/TiO_2_ model results in the formation of Cu_0.5_Pd_0.5_/TiO_2_ model with much more negative free‐energy change value (−0.29 eV) for ^*^OCCO formation, compared to that for Pd_0.5_/TiO_2_ model (−0.15 eV). The above calculation results clearly highlight the key role of Cu atom for stabilizing the critical C─C coupling intermediate (^*^OCCO) towards C_2_ product formation. Although the calculated free‐energy differences among the three catalysts appear relatively small in absolute magnitude (−0.15, −0.21, and −0.29 eV), such energy variations are of key importance in catalytic reactions. At room temperature, an energetic difference of only ∼0.1 eV corresponds to several orders of magnitude difference in the thermodynamic population and reaction probability according to the Boltzmann distribution [92, 93, 94], Therefore, the more favorable stabilization of ^*^OCCO intermediate on Cu_0.5_Pd_0.5_/TiO_2_ is expected to greatly increase the likelihood of C─C bond formation, thereby promoting the generation of C_2_ products. More importantly, the above theoretical calculation results are fully consistent with the experimental results. In situ IR measurements reveal the formation of carbonate (m‐CO_3_^2−^ and b‐CO_3_^2−^), and ^*^COOH on all catalysts (Figures 6a and S22a,b), which are common intermediates for CO_2_‐to‐CO conversion. Nevertheless, only in situ IR spectrum of Cu_0.5_Pd_0.5_/TiO_2_ (Figure 6a) exhibits the presence of C─C coupling intermediates (^*^OCCO and ^*^CH_3_COOH). Although in situ IR measurement (Figure 6a) cannot directly visualize the C─C coupling process, it convincingly reveals the apparently different surface reaction environment over Cu_0.5_Pd_0.5_/TiO_2_, compared to that over Cu_0.5_/TiO_2_ (Figure S22a) or Pd_0.5_/TiO_2_ (Figure S22b). In alignment with the above in situ IR measurement results, Cu_0.5_Pd_0.5_/TiO_2_ exhibits remarkably enhanced CH_3_COOH evolution (Figure 3a), compared to that over Cu_0.5_/TiO_2_ (Figure 3a) or Pd_0.5_/TiO_2_ (Figure 3a), indicating that the cooperative interaction between Cu and Pd sites is crucial for efficient C_2_ product formation. Notably, the proposed reaction mechanism (Figure 1) is fully consistent with the prevailing mechanistic understanding of CO_2_ reduction catalysis. Extensive experimental and theoretical calculation results reveal that Cu‐based reactive sites are uniquely capable of stabilizing adsorbed ^*^CO species and promoting subsequent C─C bond formation through ^*^CO dimerization or related coupling pathways (Table S4), whereas Pd‐based catalysts generally favor proton reduction, hydrogen evolution, or C_1_‐product formation owing to their distinct adsorption energetics (Table S4). Therefore, on basis of the afore‐mentioned experimental results, theoretical calculations and previous references, we propose a reliable reaction model, within which Cu atoms are the most probable reaction center for C─C bond formation; while neighboring Pd atom primarily contribute to charge separation, proton reduction, and offering reductive hydrogen atom. Finally, the cooperative interaction, between adjacent Cu and Pd single atoms, results in greatly enhanced activity and selectivity toward CH_3_COOH evolution over Cu_0.5_Pd_0.5_/TiO_2_. By syncing experimental data, in situ characterizations, and DFT calculations, we can propose the following mechanism for the improved photocatalytic performance for CH_3_COOH production from CO_2_ using the Cu_0.5_Pd_0.5_/TiO_2_ catalyst (Figure S25). The addition of both Pd and Cu SAs creates a synergistic effect, which significantly boosts charge carrier separation kinetics at the Cu_0.5_Pd_0.5_/TiO_2_ interface. This improvement results in a higher generation of photogenerated electrons accumulated over the Pd sites, which positively affect the adsorbed CO_2_ and drive the following multi‐electron/proton hydrogenation reactions through transferring to the Cu sites, where the C─C coupling occurs. Additionally, the energy barriers for C─C coupling reactions decrease, resulting in substantial yields and selectivity for CH_3_COOH under light exposure. The eight‐electron pathway for CO_2_ reduction to CH_3_COOH can be described through the following equations:

(1)

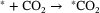



(2)

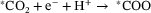



(3)

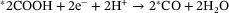



(4)

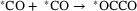



(5)

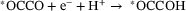



(6)

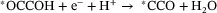



(7)

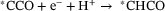



(8)

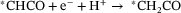



(9)

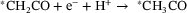



(10)

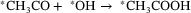



(11)

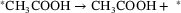




## 3 Conclusion

To conclude, we have successfully anchored Cu and Pd dual single atoms on the surface of TiO_2_ through the wet impregnation route. The synergistic effects of incorporating both Cu and Pd dual single atoms significantly enhanced acetic acid (CH_3_COOH) production for CO_2_ photoreduction in seawater environment, without any sacrificial agent and at elevated temperature (65°C), by employing a full‐spectrum light. The optimized photocatalyst Cu‐Pd dual‐single‐atom loaded TiO_2_ (Cu_0.5_Pd_0.5_/TiO_2_) exhibits a remarkable CH_3_COOH yield of 119.2 µmol/gcat with a selectivity of 84.8%. A floatable artificial leaf loaded with Cu_0.5_Pd_0.5_/TiO_2_ was engineered to maximize the gas‐liquid‐solid tri‐phase interactions, demonstrating a CH_3_COOH yield of 66.4 µmol/gcat and ethylene (C_2_H_4_) yield of 3.1 µmol/gcat after 24‐hour light irradiation.

Various in situ characterization of the photo‐induced charge kinetics within Cu_0.5_Pd_0.5_/TiO_2_ and surface reactions on Cu_0.5_Pd_0.5_/TiO_2_ surface reveal the cooperative effect of Cu‐Pd dual single atoms: (i) Pd single atoms promote the extraction of photo‐induced electrons from the conduction band of TiO_2_ and the subsequent reduction of protons to form the reactive hydrogen atoms (H·); ii) the highly reductive H· provided by the Pd single atoms can significantly boost the 8‐electron/8‐proton involved CO_2_‐to‐CH_3_COOH reaction; iii) the Cu single atoms can cooperatively works to adsorb the CO_2_ molecules and promote the C─C coupling to finally form the CH_3_COOH molecule. The density functional theory (DFT) based calculations further revealed that the presence of Cu‐Pd dual atomic sites lowers the energy barrier for the ^*^OCCO intermediate formation, favoring ^*^CO and ^*^CO coupling reactions compared to isolated Cu sites. This leads to the CH_3_COOH photogeneration pathway being more thermodynamically favored on the Cu_0.5_Pd_0.5_/TiO_2_ surface. Additionally, our photocatalytic performance assessments and control experiments reveal that photoinduced thermal effects and the seawater‐rich electrolytic content environment promote C─C coupling and hydrogen evolution, thereby improving both the yield and selectivity of CH_3_COOH production. Our findings provide valuable insights into the design of photocatalysts for the conversion of CO_2_ into high‐value C_2_ chemicals, with targeted applications.

## 4 Experimental Section

All experimental details can be found in the Supporting Information.

## Conflicts of Interest

The authors declare no conflicts of interest.

## Supporting information




**Supporting File**: adma74420‐sup‐0001‐SuppMat.docx.

## Data Availability

The data that support the findings of this study are available from the corresponding author upon reasonable request.
