# Supplementary material for: Cu‐Pd Dual Single Atoms Promoting Selective CO2 Photoreduction to C2 Products in Seawater
Source: Adv Mater. 2026 Aug 5;38(52):e74420. doi: 10.1002/adma.74420 (PMC13579086; doi:10.1002/adma.74420)
Supplement: Supplementary file 1 [file ADMA-38-e74420-s001.docx]

**Cu-Pd Dual Single Atoms Promoting Selective CO_2_ Photoreduction to C_2_ Products in Seawater**

*Elhussein M. Hashem^1^, Yiran Jiao^1^, Amin Talebian-Kiakalaieh^1^,* Xin Xu^1^, Shiying Ren^1^, Teng Liang^2^, Wenzhong Ji^3^, Teng Lu^3^, Yun Liu^3^, Bingquan Xia^4^, Ashley Slattery^5^, Jingyu Wang^6^,* Feiyan Xu^7^,* Ping She^2^,* Yan Jiao^1^, Jingrun Ran^1^**

^1^ School of Chemical Engineering, Adelaide University, Adelaide, SA 5005, Australia

^2^ State Key Laboratory of Inorganic Synthesis and Preparative Chemistry, College of Chemistry, International Center of Future Science, Jilin University, Changchun 130012, Jilin, China

^3^ Research School of Chemistry, ANU College of Science, The Australian National University, Canberra, ACT 2601, Australia

^4^ Key Laboratory of Green Chemical Engineering Process of Ministry of Education, School of Chemistry and Environmental Engineering, Wuhan Institute of Technology, Wuhan 430074, Hubei, China

^5^ Adelaide Microscopy, Adelaide University, Adelaide, SA 5005, Australia

^6^ Key Laboratory of Material Chemistry for Energy Conversion and Storage (Ministry of Education), School of Chemistry and Chemical Engineering, Huazhong University of Science and Technology, Wuhan 430074, China

^7^ Laboratory of Solar Fuel, Faculty of Materials Science and Chemistry, China University of Geosciences, Wuhan 430078, China

*Correspondence to: [amin.talebiankiakalaieh@adelaide.edu.au](mailto:amin.talebiankiakalaieh@adelaide.edu.au); [wangjingyu@hust.edu.cn](mailto:wangjingyu@hust.edu.cn); [xufeiyan@cug.edu.cn](mailto:xufeiyan@cug.edu.cn); sheping@jlu.edu.cn; [jingrun.ran@adelaide.edu.au](mailto:jingrun.ran@adelaide.edu.au)

**1. Materials**

**1.1. Preparation of Cu SAs/TiO_2_ Photocatalysts**

Cu SAs/TiO_2_ Photocatalysts were prepared via a straightforward wet impregnation method. Initially, 100 mg of commercial TiO_2_ powder (P25) was dispersed in 50 mL of deionized water and subjected to sonication for 30 minutes to ensure uniform dispersion. Subsequently, a specified volumes of Cu(NO_3_)_2_·5H_2_O solution (15.7 mM) were introduced under continuous stirring. Following an additional 30 minutes of sonication, the mixture was transferred to an oil bath and heated at 70°C with stirring for 7 hours. The resultant products were then subjected to vacuum filtration and rinsed three times with deionized water to remove excess chemicals. The filtered catalysts were allowed to dry at room temperature in a vacuum hood, after which they were stored for further characterization and application. The synthesized photocatalysts are designated as Cu_x_/TiO_2_, where x corresponds to 0.25, 0.5, 0.75, and 1 m/m%, indicating the mass percentage of Cu relative to TiO_2_.

**1.2. Preparation of Cu-Pd/TiO_2_ DSAs Photocatalysts**

A range of Cu-Pd/TiO_2_ dual single atoms (DSAs) photocatalysts were prepared using a methodology analogous to that employed for the Cu SAs catalysts on TiO_2_, incorporating several modifications. Initially, the optimized Cu_0.5_/TiO_2_ photocatalyst was prepared. Prior to the thermal treatment in the oil bath, specified volumes of a (20 mM) Na_2_PdCl_4_ solution were introduced into the Cu_0.5_/TiO_2_ precursor mixture under continuous stirring, followed by sonicating the mixture for one hour. The resultant mixture was subsequently heated at 70°C in an oil bath. Post-synthesis, the products were thoroughly washed and subjected to vacuum filtration, with rinsing performed three times using deionized water to eliminate any unreacted species. The filtered catalysts were allowed to dry at ambient conditions within a vacuum hood before being stored for subsequent characterization and application testing. The synthesized photocatalysts are referred to as Cu_0.5_Pd_x_/TiO_2_, where x denotes the mass percentage of Pd relative to TiO_2_, specifically corresponding to values of 0.2, 0.4, and 0.5 m/m%.

**1.3. Preparation of Pd_0.5_/TiO_2_ Photocatalyst**

The preparation of the Pd_0.5_/TiO_2_ photocatalyst was conducted using the same methodology employed for the fabrication of the Cu SAs/TiO_2_ photocatalyst. A specific volume of a 20 mM Na_2_PdCl_4_ solution was introduced into the suspended TiO_2_ mixture, followed by identical procedural steps. The designation "0.5" in the Pd_0.5_/TiO_2_ photocatalyst indicates the mass

percentage of Pd relative to the mass of the bare TiO_2_ support.

**1.4. Fabrication of Cu_0.5_Pd_0.5_/TiO_2_ Floatable Artificial Leaf**

The Cu_0.5_Pd_0.5_/TiO_2_ floatable artificial leaf was synthesized by initially dispersing 20 mg of the optimized Cu_0.5_Pd_0.5_/TiO_2_ composite in 5 mL of deionized water, followed by a 30-minute sonication period to ensure uniform dispersion. Subsequently, the resulting suspension was transferred into an airbrush apparatus and uniformly deposited onto a 47 mm glass fibre filter paper substrate. The coated filter paper was then allowed to dry at ambient temperature within a fume hood to facilitate proper adhesion and prepare it for subsequent applications.

**2. Physicochemical Characterization**

Powder X-ray diffraction (XRD) patterns were acquired using a Miniflex X-ray diffractometer (Rigaku) equipped with a Cu Kα radiation source. Transmission electron microscopy (TEM), high-resolution transmission electron microscopy (HRTEM), high-angle annular dark-field scanning transmission electron microscopy (HAADF-STEM), energy-dispersive X-ray spectroscopy (EDX), and EDX elemental mapping images were obtained using the FEI Titan S/TEM instrument (Thermo Fisher Scientific, USA). Atomic force microscopy (AFM) and Kelvin probe force microscopy (KPFM) images, along with corresponding height/potential profiles, were acquired using a Dimension Icon system (Bruker, USA). Inductively coupled plasma atomic emission spectroscopy (ICP-MS) was conducted using an Agilent 8900 ICP-MS instrument. X-ray photoelectron spectroscopy (XPS) characterization was carried out on a K-Alpha plus XPS system (Thermo Fisher Scientific, USA), with photocatalysts excited by a light-emitting diode in in-situ XPS measurements. UV-Vis diffuse reflectance spectra and UV-Vis absorption spectra were obtained using a UV-Vis spectrophotometer (UV2600, Shimadzu, Japan). Steady-state photoluminescence (PL) spectra at room temperature were acquired using an RF-5301PC Spectro-fluorophotometer (Shimadzu, Japan), while transient-state PL decay curves were obtained using an FLS1000 fluorescence lifetime spectrophotometer (Edinburgh Instruments, UK). In situ IR spectra were collected using a Nicolet iS20 spectrometer equipped with a HgCdTe (MCT) detector. Synchrotron-based X-ray absorption spectroscopy (XAS) measurements were collected on XAS beamline of Australian Synchrotron (ANSTO, Melbourne). Steady-state surface photovoltage (SPV) spectra were acquired using a home-built apparatus as described by Kang *et al.*^[1]^, while transient-state SPV spectra were obtained using another device introduced in the referenced work. Electron paramagnetic resonance (EPR) spectra under visible light irradiation were obtained using an EPR spectrometer (Bruker model

A300).

**3. Electrochemical Measurements**

Mott-Schottky analyses were performed using a CHI 660C electrochemical workstation configured for a standard three-electrode setup. The working electrodes, TiO_2_ and Cu_0.5_Pd_0.5_/TiO_2_, were fabricated on fluorine-doped tin oxide (FTO) coated glass substrates. A platinum wire was utilized as the counter electrode, while an Ag/AgCl reference electrode (saturated KCl) was employed. To prepare the working electrodes, we dispersed 10 mg of the active material and 15 mg of poly(ethylene glycol) (PEG; molecular weight: 20000, Sigma-Aldrich) in 1 mL of ethanol. The mixture was sonicated for 30 minutes to achieve a homogenous distribution. Subsequently, 50 µL of this suspension was drop-cast onto 0.7×1.6 cm² FTO glass electrodes using a micropipette. The coated electrodes were calcined at 350 °C in an argon atmosphere for 30 minutes in a short tube furnace. The resulting materials were then employed as the working electrodes in the electrochemical setup using a 0.5 M Na_2_SO_4_ electrolyte.

The band positions of both TiO_2_ and Cu_0.5_Pd_0.5_/TiO_2_ were calculated as follows: the valence band (VB) position was acquired according to the following equation:

$$E_{\mathrm{VB}}=E_{F}+\Delta E_{\mathrm{VB}}$$

Where $E_{F}$comes from mott-schottky (*vs.* RHE at pH = 7) and $\Delta E_{\mathrm{VB}}$is the VB edge obtained from XPS VB spectra relative to the Fermi level. Then, the conduction band (CB) position was calculated according to the following equation:

E_CB_ = E_VB_ − E_g_

E_g_ in the above equation corresponds to the optical band gap energy.

**4. Photocatalytic CO_2_ Reduction Measurements in Seawater**

The photocatalytic CO_2_ reduction experiments were carried out in a CO_2_ environment at 65°C. Typically, a total of 20 mg of the photocatalyst was dispersed in 30 mL of seawater within a 170 mL three-neck glass reactor. The seawater, collected from Glenelg Beach in Adelaide, South Australia, was vacuum-filtered to eliminate sand and microorganisms before being stored for experimentation. The reactor was sonicated for 30 minutes to ensure a homogeneous distribution of the photocatalyst. To enhance light absorption and increase the reaction temperature, the reactor was covered with a layer of aluminum foil, allowing only the upper surface to be exposed. During the initial phase, the sample suspension was stirred in the dark while laser-grade CO_2_ (99.999%) was continuously bubbled through the solution for 30 minutes, effectively saturating the seawater suspension with CO_2_. Subsequently, the reactor was irradiated with a full-spectrum 300 W xenon lamp (PLS-SXE 300, Beijing Perfectlight) for six hours, maintaining continuous stirring throughout the process. Gas products were sampled from the reactor and analyzed using gas chromatography (GC, Agilent 7890B). Concurrently, an aliquot of the reaction solution was withdrawn, filtered to remove any residual photocatalyst, and the resulting supernatant was examined using a 600 MHz ^1^H NMR spectrometer (Bruker), along with Gas Chromatography-Mass Spectrometry (GC-MS) on an Agilent 7977B/7890B. Separation was completed using a Perkin Elmer Elite-FFAP (30 m x 0.32 mm ID x 0.5 μm phase thickness) with a flow of 2 ml/min helium carrier gas. A 1μL sample was injected with an injector temperature of 250^o^C into the GC system operated in pulsed splitless mode (25psi until 0.5min and 100ml/min slit at 0.45min). The oven temperature program started at 40°C, held for one minute, followed by a 10°C/minute ramp to 150°C, then 25°C/minute ramp to 250 °C with a final hold for 4 minutes. The mass spectrometer (5977B MSD) scanned from m/z 20-300Da. The compounds were characterized with comparison to the NIST14 Spectral Library database using ChemStation MS data analysis software (Agilent). Quantification was conducted using a certified volatile-free acid mix (Supelco, CRM46975) and quantification via high-performance liquid chromatography (HPLC, Thermo Scientific RefractoMax 520). The selectivity for CO_2_ reduction into products such as CH_3_COOH, C_2_H_4_, CO, and H_2_ was calculated based on the following equations:

CH_3_COOH Sel (%) $=\frac{8\times n\left( {CH}_{3}COOH \right)}{8\times n\left( {CH}_{3}COOH \right)+2\times n\left( H_{2} \right)+2\times n\left( CO \right)+12\times n(C_{2}H_{4})}\times100\%$	(1)

C_2_H_4_ Sel (%) $=\frac{12\times n(C_{2}H_{4})}{8\times n\left( {CH}_{3}COOH \right)+2\times n\left( H_{2} \right)+2\times n\left( CO \right)+12\times n(C_{2}H_{4})}\times100\%$	(2)

CO Sel (%) $=\frac{2\times n\left( CO \right)}{8\times n\left( {CH}_{3}COOH \right)+2\times n\left( H_{2} \right)+2\times n\left( CO \right)+12\times n(C_{2}H_{4})}\times100\%$	(3)

H_2_ Sel (%) $=\frac{2\times n\left( H_{2} \right)}{8\times n\left( {CH}_{3}COOH \right)+2\times n\left( H_{2} \right)+2\times n\left( CO \right)+12\times n(C_{2}H_{4})}\times100\%$	(4)

where n is the mole amounts of the products.

The apparent quantum efficiency (AQE) of Cu_0.5_Pd_0.5_/TiO₂ for CO_2_ reduction to acetic acid was calculated by utilizing the following equation:

$AQE= \frac{8nN_{A}\mathrm{hc}}{AIt\lambda}\times100\%$	(5)

where n (7.215 μmol) is the number of moles for produced CH_3_COOH, N_A_ is the Avogadro’s constant, h is the Planck constant, c is the light velocity in vacuum, I is the intensity of the light, A (1.2 cm^2^) is the irradiation area, t is the reaction time, and λ is the wavelength of light (365 nm). Also, the AQE test was conducted utilizing 50 mg Cu_0.5_Pd_0.5_/TiO₂ dispersed in 30 mL of seawater. A 365-nm LED with a light intensity of 22.3 mW cm^−2^ was utilized as the light source for 6-hour light irradiation. The ^13^CO_2_ isotope-labelling experiments were performed to trace the carbon source of the reaction products. High-purity ^13^CO_2_ (99 atom% ^13^C, 99.9% chemical purity) was introduced into the photocatalytic reaction system in the identical conditions as those utilized for the standard CO_2_ reduction experiments. After photocatalytic reaction, the gaseous and liquid products were analyzed by utilizing a gas chromatography-mass spectrometry (GC-MS) system (8890 GC coupled with a 5977B MSD, Agilent Technologies, USA). An HP-MOLESIEVE capillary column was employed for the separation and analysis of gaseous products, while an HP-FFAP capillary column was used for the analysis of liquid products, particularly acetic acid. Helium was used as the carrier gas. The injector temperature and electron ionization (EI) source temperature were maintained at 150 and 200 °C, respectively.

**5. Computational Methods**

All density functional theory (DFT) calculations were performed using the Vienna Ab Initio Simulation Package (VASP, version 5.4.4).^[2]^ The projected augmented wave (PAW) pseudopotentials^[3]^ were employed to describe the core elections. The generalized gradient approximation (GGA) with Perdew-Burke-Ernzerhof (PBE) functional^[4]^ was used to describe the exchange-correlation potential. DFT-D3 method developed by Grimme et al.^[5]^ was applied for van der Waals corrections. The Cu_0.5_/TiO₂, Pd_0.5_/TiO_2_ and Cu_0.5_Pd_0.5_/TiO₂ models were constructed based on experimental observations and previous literature.^[6]^ Full geometric optimizations were carried out until the energy and atomic forces converged to within 10⁻⁵ eV and 0.02 eV/Å, respectively. A 15 Å vacuum layer was added perpendicular to the TiO₂ slab to eliminate interactions between periodic images. The Brillouin zone was sampled using a 3×3×1 k-point mesh, and the plane-wave energy cutoff was set to 500 eV. The Gibbs free energy change (ΔG) for each reaction step was calculated using the following equation:

ΔG = ΔE + ΔZPE – TΔS (1)

where ΔE is the DFT-calculated energy difference between products and reactants. ΔZPE and ΔS represented zero-point energy and entropy, respectively, both obtained from vibrational frequency calculations using VASPKIT.^[7]^ The temperature T was set to room temperature (298.15 K).

**Figure S1**. XRD patterns of the as prepared photocatalysts.

**Figure S2.** (a) TEM image of Cu_0.5_/TiO_2_ catalyst. (b) HAADF-STEM image of Cu_0.5_/TiO_2_ catalyst. (c) Atomic-Resolution HAADF-STEM images of Cu_0.5_/TiO_2_ from the green rectangle region in Figure S2b. (d) High-Resolution HAADF-STEM image of Cu_0.5_/TiO_2_ and the corresponding elemental mapping images of (e) Ti, (f) O, and (g) Cu elements for Cu_0.5_/TiO_2_, respectively.

**Figure S3.** (a-d) TEM images of the optimized Cu_0.5_Pd_0.5_/TiO_2_ catalyst.

**Figure S4.** (a) Atomic-Resolution HAADF-STEM image of Cu_0.5_Pd_0.5_/TiO_2_. (b) The corresponding intensity profile for atomic Pd and Cu centre along the green-coloured region 1 in Figure S4a, in which Pd atoms are circled in yellow and Cu atoms are circled in blue, respectively.

**Figure S5.** (a) The synchrotron-based XANES spectra for Cu K edge of Cu_0.5_/TiO_2_, Cu foil, Cu_2_O and CuO, respectively. Figure S5 inset exhibits the near-edge features for XANES spectra of Cu_0.5_/TiO_2_, Cu foil, Cu_2_O and CuO, respectively. (b) Fourier-transformed extended X-Ray absorption fine structure (EXAFS) spectra for Cu K edge of Cu foil, CuO, Cu_2_O and Cu_0.5_/TiO_2_. (c) Cu K-edge WT-EXAFS spectra for Cu foil, CuO, Cu_2_O and Cu_0.5_/TiO_2_.

**Figure S6.** (a) The synchrotron-based Cu K-edge XANES spectra for Cu_0.5_Pd_0.5_/TiO_2_, Cu foil, Cu_2_O and CuO, respectively. Figure S6a inset exhibits the near-edge features for XANES spectra of Cu_0.5_Pd_0.5_/TiO_2_, Cu foil, Cu_2_O and CuO, respectively. (b) Fourier-transformed extended X-Ray absorption fine structure (EXAFS) spectra for Cu foil, CuO, Cu_2_O and Cu_0.5_Pd_0.5_/TiO_2_. (c) Cu K-edge WT-EXAFS spectra for Cu foil, CuO, Cu_2_O and Cu_0.5_Pd_0.5_/TiO_2_.

**Figure S7.** High-Resolution XPS spectra of (a) Ti 2p and (b) O 1s peaks for TiO_2_ and Cu_0.5_Pd_0.5_/TiO_2_, respectively. (c) High-Resolution XPS spectra of Cu 2p peaks for Cu_0.5_Pd_0.5_/TiO_2._ (d) High-Resolution XPS spectra of Pd 3d peaks for Cu_0.5_Pd_0.5_/TiO_2_.

**Figure S8.** The EPR spectrum of Cu_0.5_Pd_0.5_/TiO_2_.

**Figure S9.** ^1^HNMR spectrum for the collected seawater before photocatalytic experiments.

**Figure S10.** ^1^H NMR spectra for (a) Reference acetic acid, (b) the liquid product generated by Cu_0.5_Pd_0.5_/TiO_2_ after 6 h test in seawater, and (c) the liquid product generated by Cu_0.5_Pd_0.5_/TiO_2_ after 24 h test in seawater.

**Figure S11.** (a) GC-MS chromatogram result of the liquid product generated by Cu_0.5_Pd_0.5_/TiO_2_ after a 6-h test in seawater. (b) Mass spectrum of acetic acid obtained from Cu_0.5_Pd_0.5_/TiO_2_ after a 6-h test in seawater. (c) GC-MS chromatogram result of the liquid product generated by Cu_0.5_Pd_0.5_/TiO_2_ after a 24-h test in seawater. (d) Mass spectrum of acetic acid obtained from Cu_0.5_Pd_0.5_/TiO_2_ after a 24-h test in seawater.

**Figure S12.** HPLC results for (a) Reference acetic acid, (b) the liquid product generated by Cu_0.5_Pd_0.5_/TiO_2_ after a 6-h test in seawater, and (c) the liquid product generated by Cu_0.5_Pd_0.5_/TiO_2_ after a 24-h test in seawater.

**Figure S13.** GC-MS chromatography results of (a) produced ^13^CH_3_^13^COOH (b) produced ^13^CO via ^13^CO_2_ photoreduction over Cu_0.5_Pd_0.5_/TiO_2_.

**Figure S14.** UV-Vis diffuse Reflectance spectra of as-prepared photocatalysts and corresponding photographs.

**Figure S15.** Mott-Schottky plots of (a) TiO_2_ and (b) Cu_0.5_Pd_0.5_/TiO_2_, respectively.

**Figure S16.** XPS valence band (VB) spectra of (a) TiO_2_ and (b) Cu_0.5_Pd_0.5_/TiO_2_, respectively.

**Figure S17.** The schematic diagram for electronic band structures of TiO_2_ and Cu_0.5_Pd_0.5_/TiO_2_, respectively.

**Figure S18.** The steady-state PL spectra of TiO_2_, Cu_0.5_/TiO_2_, Pd_0.5_/TiO_2_ and Cu_0.5_Pd_0.5_/TiO_2_.

**Figure S19.** In situ high resolution XPS spectra of (a) Ti 2p and (b) O 1s peaks for the bare TiO_2_ under the following three different conditions: i) in the dark and vacuum condition; ii) under light irradiation and in vacuum condition; and iii) under light irradiation and in CO_2_ atmosphere (light + CO_2_), respectively.

**Figure S20.** In situ EPR spectra for bare TiO_2_ with light on and off in a CO_2_ atmosphere, respectively.

**Figure S21.** (a) AFM image and (b) the corresponding height profile of Cu_0.5_Pd_0.5_/TiO_2_ in the dark condition. (c) AFM image and (d) the corresponding height profile of Cu_0.5_Pd_0.5_/TiO_2_ with light irradiation.

**Figure S22**. (a) In situ IR spectra for the CO_2_ PR over Cu_0.5_/TiO_2_ and (b) Pd_0.5_/TiO_2_ in the presence of CO_2_ and at a temperature of 65 °C.

**Figure S23**. The theoretical models for the optimized (a) Pd_0.5_/TiO_2,_ (b) Cu_0.5_/TiO_2_ and (c) Cu_0.5_Pd_0.5_/TiO_2_ configurations, respectively.

**Figure S24**. The calculated Gibbs free energy diagram for CO_2_-to-CH_3_COOH conversion via C-C coupling over Pd_0.5_/TiO_2_ model.

**Figure S25**. The schematic diagram for the proposed mechanism of CO_2_ PR into acetic acid over the optimized Cu_0.5_Pd_0.5_/Ti

**Table S1.** Curve fit parameters ^a^ for Cu K-edge EXAFS results for Cu_0.5_Pd_0.5_/TiO_2_.

| Path | d^b^/Å | CN | R/Å | σ^2^/Å^2^ |
| --- | --- | --- | --- | --- |
| Cu-O | 1.952 | 3 | 1.97±0.01 | 0.004±0.001 |

^a^ S_0_^2^ was fixed as 0.9. ∆*E_0_* was refined as a global fit parameter, returning as value of (1±2) eV. Data ranges: 2.5 ≤ *k* ≤ 11 Å^-1^, 1.0 ≤ R ≤ 3.0 Å. The number of variable parameters is 7, out of a total of 10.6 independent data points. R factor for this fit is 2.5 %. ^b^ The distance for Cu-O is from the crystal structure of CuO.

| **Ions** | **Concentration (mg/L)** |
| --- | --- |
| Ca^2+^ | 507 |
| Cl^-^ | 19000 |
| K^+^ | 478 |
| Mg^2+^ | 1720 |
| Na^+^ | 14000 |
| SO_4_^2-^ | 3300 |
| S^2-^ | 1100 |

**Table S2**. Ion analysis for the collected seawater utilizing ICP-MS for metal analysis and a discrete analyzer for chloride analysis.

**Table S3**. A comparison of photocatalytic CO_2_ reduction to acetic acid in the reported references and in our work.

| **Entry** | **Catalyst** | **Catalyst amount (mg)** | **Light source** | **Reaction condition** | **Reaction time**  **(h)** | **Acetic Acid yield**  **(µmol g^-1^ h^-1^)** | **Acetic Acid selectivity**  **(%)** | **Reference** |
| --- | --- | --- | --- | --- | --- | --- | --- | --- |
| 1 | Cu/WO_3_ | 40 | 300 W Xe lamp | 40 mg catalyst + 20 mL DI water in a 700 mL glass reactor with quartz window + CO_2_ bubbling for 40 min + reaction temperature kept at 15 ℃ + light irradiation | 2 | 2.87 | 67 | [8] |
| 2 | H-WO_3_ | 50 | solar simulator (PEC-L11) | 50 mg catalyst + 150 mL DI water in a 600 mL pyrex reactor with quartz window + CO_2_ (50% in Ar) bubbling for 30 min + reaction temperature kept at 4 ℃ + light irradiation | 10 | 9.4 | 85 | [9] |
| 3 | NiCo-TiO_2_ | 20 | 300 W Xe lamp | 20 mg catalyst + 50 mL DI water + 0.1 M Na_2_SO_3_ + 0.2 M CsOH + CO_2_ (99.999%) bubbling for 30 min + reaction temperature kept at Room temperature + light irradiation | N/A | 22.6 | 71 | [10] |
| 4 | 3%Nb_2_O_5_/TNTs | N/A | 300 W Xe lamp | catalyst + 20 mL DI water in a 30 mL reactor + CO_2_ bubbling (10 mL/min) in test+ reaction temperature kept at ambient condition + light irradiation | 4 | 28.4 | 69 | [11] |
| 5 | Vo-rich Zn_2_GeO_4_ | 10 | Xe lamp (100 mW/cm^2^, AM 1.5 G filter) | 10 mg catalyst coated over a quartz plate + 12 mL DI water + vacuum-treated + bubbling CO_2_ (0.03 vol% CO_2_/Ar) until reach 1 atmosphere | 10 | 12.7 | 66.9 | [12] |
| 6 | Cu^0^-TiO_2_ | 100 | UVC lamp (254 nm) | 100 mg catalyst + 100 mL sodium oxalate (0.1 mol/L) in a 150 mL quartz tube + CO_2_ bubbling for 10 min + reaction temperature kept at 15 ℃ + light irradiation | 6 | 17.58 | 28.6 | [13] |
| 7 | partially Reduced Co_3_O_4_ NSs | 2.5 | 300 W Xe lamp (100 mW/cm^2^, AM 1.5 G filter) | 2.5 mg catalyst coated on quartz dish + 5 mL DI water + simulated air (0.03% CO_2_ + 79.98% N_2_ + 19.99% O_2_) + reaction temperature kept at 25 ℃ + light irradiation | 5 | 2.95 | 92.5 | [14] |
| 8 | Pd-Co_3_O_4_ | 3 | 300 W Xe lamp (100 mW/cm^2^, AM 1.5 G filter) | 3 mg catalyst coated uniformly onto a homemade quartz glass and heated at 60 ℃ + CO_2_ bubbling (99.99%) + light irradiation | 2 | 13.8 | 100 | [15] |
| 9 | FeOOH-Bi_2_MoO_6_ | 10 | 300 W Xe lamp (100 mW/cm^2^, AM 1.5 G filter) | 3 mg catalyst coated uniformly onto a homemade quartz glass and heated at 60 ℃ + CO_2_ bubbling (99.99%) + light irradiation | 6 | 5.5 | 79.3 | [16] |
| 10 | Nb/Cu-2.5% | 300 | UVC lamp (OSRAM 11 W, 253.7 nm, 21.49 mW/cm^2^) | 50 mg catalyst + 300 mL DI water in a 500 mL borosilicate glass reactor + CO_2_ bubbling for 20 min + light irradiation | 24 | 3 | 54.6 | [17] |
| 11 | Cu_0.5_Pd_0.5_ /TiO₂ | 20 | 300 W Xe lamp | 20 mg catalyst + 30 mL seawater in a 170 mL three-neck glass reactor covered with aluminium foil except for the top side + CO_2_ bubbling 99.999% for 30 min + temperature naturally kept at 65 ℃ by light irradiation + light irradiation | 6 | 19.8 | 84.8 | Our work |

**Table S4.** Role of Cu and Pd sites in CO_2_ photocatalytic Conversion.

| Catalyst | Reactive Site | Role | Ref |
| --- | --- | --- | --- |
| Au_0.2_Cu_0.3_Bi_2_WO_6_ | Cu SAs | C–C coupling | ^[18]^ |
| Cu-Ti-V_O_/Ti_0.91_O_2_-SL | Cu SAs | Reducing the energy levels of the key intermediates and C–C coupling | ^[19]^ |
| Cu/3DOM-In_2_O_3_ | Cu SAs | Promote formation of the key intermediates and C–C coupling | ^[20]^ |
| CuACs/PCN | Cu SAs | Reducing the energy barrier in stepwise reactions and C–C coupling | ^[21]^ |
| Cu/S-RPCN | Cu SAs | Promote formation of the key intermediates and C–C coupling | ^[22]^ |
| Pd_1+NPs_/C_3_N_4_ | Pd SAs and Pd NPs | Boosting H_2_O dissociation for H* Formation and promoting multiple proton–electron coupling transfer | ^[23]^ |
| Pd_NPs_/TiO_2_ | Pd NPs | CO_2_ hydrogenation | ^[24]^ |
| Pd_x_Cu_y_/ZnO-CN | Pd SAs | Internal (Pd→Cu) electron transfer and facilitating the formation of *CHCHO and *CH_2_CO intermediates | ^[25]^ |
| Pd_2_Cu NPs/P25 | Pd NPs | Enhance charge transfer to Cu and promote CO_2_ hydrogenation | ^[26]^ |

**References**

[1]	C. Kang, L. Jing, T. Guo, H. Cui, J. Zhou, H. Fu, *J. Phys. Chem. C* **2009**, *113*, 1006.

[2]	G. Kresse, J. Furthmüller, *Comput. Mater. Sci.* **1996**, *6*, 15.

[3]	D. Joubert, *Phys. Rev. B - Condens. Matter Mater. Phys.* **1999**, *59*, 1758.

[4]	J. P. Perdew, K. Burke, M. Ernzerhof, *Phys. Rev. Lett.* **1996**, *77*, 3865.

[5]	S. Grimme, J. Antony, S. Ehrlich, H. Krieg, *J. Chem. Phys.* **2010**, *132*, DOI 10.1063/1.3382344.

[6]	C. Ren, Q. Li, C. Ling, J. Wang, *J. Am. Chem. Soc.* **2023**, *145*, 28276.

[7]	V. Wang, N. Xu, J. C. Liu, G. Tang, W. T. Geng, *Comput. Phys. Commun.* **2021**, *267*, 108033.

[8]	D. Zeng, H. Wang, X. Zhu, H. Cao, Y. Zhou, W. Wang, L. Zhang, W. Wang, *Chem. Eng. J.* **2023**, *451*, 138801.

[9]	S. Sun, M. Watanabe, J. Wu, Q. An, T. Ishihara, *J. Am. Chem. Soc.* **2018**, *140*, 6474.

[10]	G. Jia, M. Sun, Y. Wang, Y. Shi, L. Zhang, X. Cui, B. Huang, J. C. Yu, *Adv. Funct. Mater.* **2022**, *32*, 1.

[11]	R. Lv, K. Liu, H. Hu, M. Fan, K. Li, M. Zhang, H. Huang, *Sep. Purif. Technol.* **2025**, *359*, 130835.

[12]	J. Zhu, W. Shao, X. Li, X. Jiao, J. Zhu, Y. Sun, Y. Xie, *J. Am. Chem. Soc.* **2021**, *143*, 18233.

[13]	J. A. Torres, J. C. da Cruz, A. E. Nogueira, G. T. S. T. da Silva, J. A. de Oliveira, C. Ribeiro, *J. Environ. Chem. Eng.* **2022**, *10*, DOI 10.1016/j.jece.2022.107291.

[14]	S. Zhu, X. Li, X. Jiao, W. Shao, L. Li, X. Zu, J. Hu, J. Zhu, W. Yan, C. Wang, Y. Sun, Y. Xie, *Nano Lett.* **2021**, *21*, 2324.

[15]	J. Ding, P. Du, J. Zhu, Q. Hu, D. He, Y. Wu, W. Liu, S. Zhu, W. Yan, J. Hu, J. Zhu, Q. Chen, X. Jiao, Y. Xie, *Angew. Chemie - Int. Ed.* **2024**, *63*, DOI 10.1002/anie.202400828.

[16]	D. He, G. Huang, J. Hu, J. Ding, W. Liu, L. Chen, W. Yan, J. Zhu, S. Zhu, Q. Chen, X. Jiao, Y. Xie, *Adv. Energy Mater.* **2025**, *15*, 1.

[17]	A. E. Nogueira, G. T. S. T. Silva, J. A. Oliveira, O. F. Lopes, J. A. Torres, M. Carmo, C. Ribeiro, *ACS Appl. Energy Mater.* **2020**, *3*, 7629.

[18]	B. Liu, M. Cheng, C. Zhang, Y. Si, J. Zhou, Y. Ren, J. Guan, L. Duan, M. Liu, D. Jing, N. Li, *Appl. Catal. B Environ.* **2024**, *357*, 124263.

[19]	W. Li, X. shan Chu, F. Wang, Y. yan Dang, X. yun Liu, T. hao Ma, J. yuan Li, C. yi Wang, *Appl. Catal. B Environ.* **2022**, *304*, 121000.

[20]	M. Xu, Q. Zhang, S. Wei, S. Liu, M. Zhou, Y. Zhao, B. Li, Y. Xie, *Angew. Chemie - Int. Ed.* **2025**, *64*, DOI 10.1002/anie.202506072.

[21]	W. Xie, K. Li, X. H. Liu, X. Zhang, H. Huang, *Adv. Mater.* **2023**, *35*, 1.

[22]	W. Li, Z. Liu, B. Rhimi, M. Zhou, J. Li, K. Nie, B. Yan, Z. Jiang, W. Shi, Y. Xiong, *Angew. Chemie - Int. Ed.* **2025**, *64*, DOI 10.1002/anie.202423859.

[23]	P. Liu, Z. Huang, X. Gao, X. Hong, J. Zhu, G. Wang, Y. Wu, J. Zeng, X. Zheng, *Adv. Mater.* **2022**, *34*, 1.

[24]	Z. Yang, T. Zhao, Y. Tang, Y. Jiang, H. Kitagawa, X. Wen, F. Wang, *J. Catal.* **2023**, *424*, 22.

[25]	G. Wang, H. Su, S. Chen, Y. Wang, Z. Chen, F. Chen, H. Yin, J. Li, *Appl. Catal. B Environ.* **2025**, *366*, 124997.

[26]	S. Bai, Q. Shao, P. Wang, Q. Dai, X. Wang, X. Huang, *J. Am. Chem. Soc.* **2017**, *139*, 6827.
